# A Forward Model Incorporating Elevation-Focused Transducer Properties for 3-D Full-Waveform Inversion in Ultrasound Computed Tomography

**DOI:** 10.1109/TUFFC.2023.3313549

**Published:** 2023-10-17

**Authors:** Fu Li, Umberto Villa, Nebojsa Duric, Mark A. Anastasio

**Affiliations:** Department of Bioengineering, University of Illinois at Urbana-Champaign, Urbana, IL 61801 USA; Oden Institute, The University of Texas at Austin, Austin, TX 78712 USA; Department of Imaging Sciences, University of Rochester, Rochester, NY 14642 USA.; Department of Bioengineering, University of Illinois at Urbana-Champaign, Urbana, IL 61801 USA

**Keywords:** 3-D full-waveform inversion (FWI), lens-focused transducer, ultrasound computed tomography (USCT)

## Abstract

Ultrasound computed tomography (USCT) is an emerging medical imaging modality that holds great promise for improving human health. Full-waveform inversion (FWI)-based image reconstruction methods account for the relevant wave physics to produce high spatial resolution images of the acoustic properties of the breast tissues. A practical USCT design employs a circular ring-array comprised of elevation-focused ultrasonic transducers, and volumetric imaging is achieved by translating the ring-array orthogonally to the imaging plane. In commonly deployed slice-by-slice (SBS) reconstruction approaches, the 3-D volume is reconstructed by stacking together 2-D images reconstructed for each position of the ring-array. A limitation of the SBS reconstruction approach is that it does not account for 3-D wave propagation physics and the focusing properties of the transducers, which can result in significant image artifacts and inaccuracies. To perform 3-D image reconstruction when elevation-focused transducers are employed, a numerical description of the focusing properties of the transducers should be included in the forward model. To address this, a 3-D computational model of an elevation-focused transducer is developed to enable 3-D FWI-based reconstruction methods to be deployed in ring-array-based USCT. The focusing is achieved by applying a spatially varying temporal delay to the ultrasound pulse (emitter mode) and recorded signal (receiver mode). The proposed numerical transducer model is quantitatively validated and employed in computer simulation studies that demonstrate its use in image reconstruction for ring-array USCT.

## Graphical Abstract

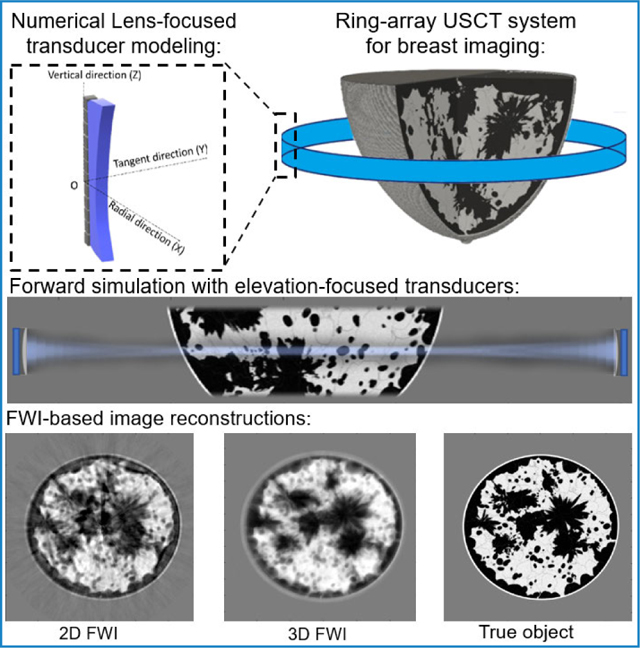

## I. Introduction

Ultrasound computed tomography (USCT) is an emerging medical imaging technology that is being developed for a variety of clinical applications [1], [2], [3], [4], [5], [6]. For example, USCT methods for cancer detection and treatment response monitoring have been reported [1], [5], [7], [8], [9], [10], [11]. Images that represent accurate estimates of the speed of sound (SOS), density, and acoustic attenuation (AA) of tissue can be produced by use of USCT image reconstruction methods [12], [13], [14], [15], [16]. To account for the relevant wave physics and thereby achieve high spatial resolution images, full-waveform inversion (FWI) reconstruction methods [3], [13], [17], [18] are being developed. Such advanced reconstruction methods can circumvent the limitations of simplified physics methods [19], [20].

Recently, a variety of 3-D USCT measurement configurations have been proposed [3], [10], [21], [22], [23], [24], [25], [26], [27], [28], [29], [30]. For example, a 3-D USCT system utilizing a hemispherical array of transducers was developed by Stotzka et al. [26], Gemmeke et al. [27]. A system developed by QT Ultrasound (QT Ultrasound, Inc., Novato, CA, USA) employs a plane wave transmitter and a 2-D flat detector [10], [28] to achieve 3-D imaging.

Another practical USCT design that has been employed by multiple groups involves a circular ring-array containing elevation-focused transducer elements [24], [25], [31], [32], [33], [34], [35]. A few examples that employ this configuration include the SoftVue system (Delphinus Medical Technologies, Inc., Novi, MI, USA), approved by the Food and Drug Administration for the screening of women with dense breast tissue and diagnostic use for all women [7], the UltraLucid system developed by Song et al. [25], [33], and a brain imaging system developed by Guasch et al. [3], [29], [30]. The ring-array system enables acquisition of ultrasound measurements at multiple vertical positions by translating the ring of transducers vertically. This design allows for sliceby-slice (SBS) image reconstruction, where a 3-D volume is obtained by stacking together 2-D slices reconstructed from USCT measurements acquired at each vertical position of the ring-array [13], [14], [36].

While the SBS reconstruction approach for ring-array-based systems alleviates the computational burden associated with directly reconstructing a 3-D volume, it possesses significant limitations. First and foremost, artifacts may appear in the reconstructed images due to scattering and diffraction from out-of-plane structures. Such effects are not accounted for in the 2-D imaging model [10], [36], where point-like transducers are typically assumed and the spatial impulse responses (SIRs) of transducers are not modeled [37], [38]. Moreover, there are also significant differences in the way acoustic waves propagate in 2-D compared to 3-D. To reduce artifact levels and improve image quality in ring-array-based USCT, there remains an important need to develop reconstruction methods based on 3-D wave physics models that can incorporate the elevational focusing properties of the transducers.

In this study, a 3-D USCT forward model that accounts for the transducer’s elevational focusing characteristics is developed to enable improved FWI-based image reconstruction in ring-array-based USCT. In this way, the out-of-plane acoustic scattering and the focusing properties (i.e., the SIRs) of the transducers, in both transmit and receive modes, are accounted for in the forward and associated adjoint models that are utilized by image reconstruction methods. Specifically, a rigid flat transducer element coupled with a concave acoustic lens is considered. The incorporation of focusing effects induced by an acoustic lens in a numerical wavesolver-based USCT forward model is the main contribution of this work. This differs from related works [39], [40] that are mostly concerned with accurate modeling of transducers with finite curved apertures that may not conform to the computational grid.

The proposed forward model is systematically validated by the use of an analytic solution. Additionally, the developed forward and adjoint models are utilized in a numerical case study of ring-array-based USCT that employ realistic 3-D numerical breast phantoms (NBPs). This demonstrates, for the first time, the feasibility of incorporating transducer elevational focusing effects in a 3-D time-domain FWI reconstruction method for ring-array-based USCT and reveals improvements in image quality over the traditional SBS reconstruction approach. To enable related investigations by other researchers, the 3-D NBPs and corresponding 3-D USCT measurement data used in the case study have been made publicly available under CC-0 licensing [41].

The remainder of this article is organized as follows. In Section II, a brief review of the canonical USCT forward model and the time-domain FWI method is provided, as well as background on ring-array-based USCT and lens-focused transducer modeling. Section III introduces the proposed USCT forward model that incorporates elevational focusing effects. The forward model is validated and characterized as reported in Section IV. A case study is presented in Section V to demonstrate the utility of the proposed forward and adjoint models for 3-D FWI. Section VI provides discussions of the numerical results presented in the case study, as well as of the limitations and future developments of the proposed approach. Finally, a summary of the work is provided in Section VII.

## II. Background

### A. Canonical Forward Models for USCT

USCT imaging models in their continuous-to-continuous (C-C) form and discrete-to-discrete (D-D) forms are reviewed below. In USCT, a sequence of short acoustic pulses are transmitted from transducer emitters and subsequently propagate through an object. The acoustic pulse is denoted as $x(t)\in L^{2}([0,T])$ where t∈[0,T] is the time coordinate, and T is a fixed finite time interval. The spatiotemporal source $s_{i}(r,t)\in L^{2}\left({\mathbb{R}}^{3}\times [0,T]\right)$ that excites the ith transducer is defined as $s_{i}(r,t)={ℰ}_{i}x(t)\equiv \chi_{i}(r)x(t)$ for i=0,1,...,M−1. Here, ${ℰ}_{i}:L^{2}([0,T])\mapsto L^{2}\left({\mathbb{R}}^{3}\times [0,T]\right)$ is the mapping operator, $r\in {\mathbb{R}}^{3}$ denotes the spatial coordinate, $\chi_{i}(r)$ is the indicator function describing the support of the active area of the ith transducer surface, and M is the total number of emitting transducers.

When the ith source pulse $s_{i}(r,t)$ propagates through the object, it generates a pressure wavefield distribution denoted by $p_{i}(r,t)\in {\text{ℍ}}^{1}\left({\mathbb{R}}^{3}\times [0,T]\right)$.^1^ The wave propagation in heterogeneous media can be described by the following lossy second-order wave equation [44]:

(1) $$\{\begin{array}{l} \frac{1}{c^{2}}\frac{\partial^{2}}{\partial t^{2}}p_{i}-\rho \nabla \cdot \frac{1}{\rho }\nabla p_{i}+L\nabla^{2}p_{i}=s_{i}\\ L=\mu \frac{\partial }{\partial t}{\left(-\nabla^{2}\right)}^{\frac{y}{2}-1}+\eta {\left(-\nabla^{2}\right)}^{\frac{y-1}{2}} \end{array}$$

where $p_{i}=p_{i}(r,t)$, $s_{i}=s_{i}(r,t)$, and c=c(r), α=α(r), ρ=ρ(r) denote the heterogeneous SOS, AA, and density distributions, respectively. The pseudo-differential operator L models power-law frequency-dependent acoustic absorption and dispersion using fractional Laplacians. In particular, the first term stems from the Chen and Holm’s [45] reformulation of Szabo’s [46] causal convolution operator and the second term is a dispersion correction derived from the Kramers–Kronig relations [47]. The quantities μ and η denote absorption and dispersion proportionality coefficients, that are defined as $\mu (r)=-2\alpha (r)c{\left(r\right)}^{y-1}$ and $\eta (r)=2\alpha (r)c{\left(r\right)}^{y}\tan(\pi y/2)$, where y=y(r) is power-law exponent. Utilizing the fractional derivative enables the employment of noninteger power-law order y and the fractional Laplacian operator enables efficient implementation through time-domain pseudospectral methods [44].

Equation (1) can be expressed in operator form as

(2) $$p_{i}(r,t)={ℋ}^{u(r)}s_{i}(r,t)$$

where $s_{i}(r,t):={ℰ}_{i}x(t)$. The linear operator ${ℋ}^{u(r)}:L^{2}({\mathbb{R}}^{3}\times [0,T])\to {\text{ℍ}}^{1}\left({\mathbb{R}}^{3}\times [0,T]\right)$ describes the action of the wave equation and has an explicit dependence on the distribution of acoustic properties u(r)=[c(r),α(r),ρ(r),y(r)].

During data acquisition, $p_{i}(r,t)$ is only recorded at a limited number of transducer receivers. These measurement data will be referred to as $g_{ij}(t)\in L^{2}([0,T])$, with the subscript i denoting that the data were produced by the ith source, also referred to as the ith data acquisition, and the subscript j denoting the data recorded at the jth transducer for j=0,1,...,M−1. In the case where discrete sampling effects and transducer focusing properties are not considered, the idealized C-C forward modelcan therefore be described as

(3) $$g_{ij}(t)={ℳ}_{j}{ℋ}^{u(r)}s_{i}(r,t)\;\text{with}\;s_{i}(r,t)={ℰ}_{i}x(t).$$

For all emitters i=0,1,...,M−1 and all receivers j=0,1,...,M−1. Here, ${ℳ}_{j}:{\text{ℍ}}^{1}\left({\mathbb{R}}^{3}\times [0,T]\right)\mapsto L^{2}([0,T])$ extracts the measurements corresponding to the jth transducer location on the measurement aperture; specifically, $g_{ij}(t)={ℳ}_{j}p_{i}(r,t)\equiv \int_{{\mathbb{R}}^{3}}\chi_{j}(r)p_{i}(r,t)d\;r$. Note that, because of acoustic reciprocity, the mapping operator ${ℰ}_{i}$ of the ith transducer is the adjoint of the sampling operator ${ℳ}_{i}$, i.e., ${ℰ}_{i}={ℳ}_{i}^{†}$.

The description of a digital imaging system is typically approximated in practice by a D-D imaging model. To establish this, u(r), x(t), $s_{i}(r,t)$, $p_{i}(r,t)$, $g_{ij}\left(t\right)$ are sampled on a Cartesian grid and at a temporal interval Δt to obtain the finite-dimensional representations as $u\in {\mathbb{R}}^{4K}$, $x\in {\mathbb{R}}^{L}$, $s_{i}\in {\mathbb{R}}^{KL}$, $p_{i}\in {\mathbb{R}}^{KL}$, and $g_{ij}\in {\mathbb{R}}^{L}$. Here, K and L denote the number of spatial and temporal samples, respectively. Let ${\left[z\right]}_{k}$ denote the kth element of a vector z. These discretized parameters can be defined as

$${\left[u\right]}_{k}:=c\left(r_{k}\right),\;{\left[u\right]}_{K+k}:=\alpha \left(r_{k}\right),\;{\left[u\right]}_{2K+k}:=\rho \left(r_{k}\right)$$

$${\left[u\right]}_{3K+k}:=y\left(r_{k}\right),\;{\left[x\right]}_{l}:=x\left(t_{l}\right),\;{\left[s_{i}\right]}_{kL+l}:=s_{i}\left(r_{k},t_{l}\right)$$

$${\left[p_{i}\right]}_{kL+l}:=p_{i}\left(r_{k},t_{l}\right),\;\text{and}\;{\left[g_{ij}\right]}_{l}:=g_{ij}\left(t_{l}\right)$$

for k=0,1,…,K−1, l=0,1,…,L−1, where $r_{k}$ denotes the kth spatial grid point and $t_{l}=l\text{Δ}t$ denotes the lth time sample. Given these discretized quantities, a D-D version of the idealized imaging model in (3) can be expressed as

(4) $$g_{ij}=M_{j}H^{\text{u}}s_{i}\;\text{with}\;s_{i}=M_{i}^{⊤}x.$$

For all emitters i=0,1,...,M–1 and all receivers j=0,1,...,M−1. Above, the matrix $H^{u}\in {\mathbb{R}}^{KL\times KL}$ represents a discrete approximation of the wave propagation operator ${ℋ}^{u(r)}$, whose action is implemented by use of a numerical wave solver method. The matrix $M_{j}\in {\mathbb{R}}^{L\times KL}$ denotes the discretization of the sampling operators ${ℳ}_{j}$ corresponding to the jth transducer, and the superscript ${\text{.}}^{⊤}$ denotes the transpose operation. Specifically, $M_{j}={\tilde{M}}_{j}⊗I_{L}$, where ${\tilde{M}}_{j}\in {\mathbb{R}}^{K\times 1}$ stems from the discretization of the indicator function $\chi_{j}(r)$ (i.e., ${\left[{\tilde{M}}_{j}\right]}_{k}=\chi_{j}\left(r_{k}\right)$ for k=0,1,…,K−1), $I_{L}\in {\mathbb{R}}^{L\times L}$ is the L-dimensional identity matrix, and ⊗ denotes the Kronecker product. In the special case where the jth transducer is point-like and located at the position $r_{k}$, the entries of the matrix ${\tilde{M}}_{j}$ are all zero but the k-row entry that is equal to 1.

Finally, by collecting in a single vector $g_{i}\in {\mathbb{R}}^{ML}$, the measurement data $g_{ij}$ recorded by all receivers when the ith emitter is excited, the discrete imaging model in (4) can be rewritten in a more compact form as

(5) $$g_{i}=MH^{\text{u}}s_{i}$$

where $M\in {\mathbb{R}}^{ML\times KL}$ is defined as $\left[M_{0};M_{1};...;M_{M-1}\right]$, and $g_{i}\in {\mathbb{R}}^{ML}$ is defined as $g_{i}=\left[g_{i0};g_{i1};\ldots ;g_{i(M-1)}\right]$. Here, the semicolon notation denotes row-wise concatenation.

### B. Time-Domain Waveform Inversion With Source Encoding (WISE) in Its Discrete Form

The USCT reconstruction problem is to estimate the tissue acoustic properties u, or a subset of them, from a collection of (noisy) measurement data $\underline{g_{i}}\in {\mathbb{R}}^{ML}$, for i=0,1,…,M−1. The underlined notation denotes that $\underline{g_{i}}$ is measured data as opposed to the simulated data $g_{i}$ produced by use of the forward model. This problem can be solved by use of the WISE method [13], [48], [49]. The WISE method is a FWI method that circumvents the large computational burden of conventional FWI [13], [18] by leveraging the superposition of acoustic waves corresponding to the excitation of multiple emitters. When an $\ell^{2}$-norm is employed as the misfit functional, the WISE method can be formulated as a stochastic optimization problem as [13]

(6) $$\hat{u}=\arg\underset{u}{\min}E_{w}\frac{1}{2}{\left\|\underline{g^{w}}-MH^{\text{u}}s^{w}\right\|}_{2}^{2}+\lambda R(u)$$

where $w\in {\mathbb{R}}^{M}$ is a random encoding vector with zero mean and identity covariance matrix, $E_{\text{w}}$ denotes the expectation with respect to w, λ is a regularization parameter, and R(u) is a regularization penalty. The quantities $\underline{g^{w}}=\sum_{i=0}^{M-1}{\left[w\right]}_{i}\underline{g_{i}}$ and $s^{w}={\sum_{i=0}^{M-1}\left[w\right]}_{i}s_{i}={\sum_{i=0}^{M-1}\left[w\right]}_{i}M_{i}^{⊤}x$ are the encoded measurement data and the encoded source, respectively. In previous studies, the encoding vector w has been chosen according to a Rademacher distribution [13], [50].

### C. Modeling of Lens-Focused Transducers Based on Geometrical Acoustics Approximation

In ring-array USCT systems, an elevational focusing effect can be achieved by the use of an acoustic lens attached to the front face of a flat transducer element [32], [51], [52]. The variation in the thickness of a concave lens introduces a spatially dependent time delay in the wavefield upon propagation through the lens.

One approach to modeling a flat transducer is based on the Rayleigh–Sommerfeld integral [53], [54]. In transmit mode, the generated acoustic pressure can be described as the superimposition of the contributions of point sources that span the transducer aperture $\text{Ω}\subset {\mathbb{R}}^{3}$, which describes the active area of the transducer element. An acoustic source on an aperture may be modeled as a velocity source or a pressure source. When an aperture surface Ω of an otherwise perfectly rigid boundary vibrates with a normal velocity $v_{⊥}(r,t)$, the acoustic pressure p(r,t) radiated from Ω, without a lens present, into a homogeneous nonlossy medium with constant SOS $c_{0}$ and density $\rho_{0}$ can be analytically described as

(7) $$p(r,t)=\rho_{0}\iint_{\text{Ω}}\frac{1}{2\pi \left\|r-r^{\prime }\right\|}\frac{\partial }{\partial t}v_{⊥}\left(r^{\prime },t-\frac{\left\|r-r^{\prime }\right\|}{c_{0}}\right)dr^{\prime }.$$

Equation (7) can be generalized to the case where a lens is attached to the transducer by incorporating a time delay into the normal velocity term. One way to accomplish this is to invoke a geometrical acoustics approximation to describe the interaction of the pressure wavefield with the lens, and neglect any effects associated with mode conversion into shear waves and refraction at the lens surface [55], [56], [57], [58], [59], [60]. The impacts associated with attenuation of the lens and impedance mismatches between the lens and propagation medium can be modeled by introducing an apodization weights function, which is discussed in Appendix B but omitted in this section.

In this case, the transducer properties introduced by the curvature of the lens can be modeled implicitly by defining the normal component of the velocity as

(8) $$v_{⊥}\left(r^{\prime },t\right)=\hat{v}\left(t+\tau \left(r^{\prime }\right)\right)\;\forall r^{\prime }\in \text{Ω}.$$

Here, the quantity $\hat{v}(t)$ denotes the normal velocity of the transducer element front face that is constant in space over the aperture Ω, and $\tau \left(r^{\prime }\right)$ is a spatially varying time shift. The time shift is determined by the thickness profile $d\left(r^{\prime }\right)$ and SOS, $c_{\text{lens}}$, of the concave lens as [57], [58], [59]

(9) $$\tau \left(r^{\prime }\right)=d\left(r^{\prime }\right)\left(\frac{1}{c_{0}}-\frac{1}{c_{\text{lens}}}\right).$$

In receive mode, the recorded signal is affected by the finite detecting aperture, again referred to as Ω, and the time delays introduced by the lens mounted on it [61]. In this case, the measured signal $g_{\text{Ω}}(t)$ can be analytically described by an integral of the incident pressure wavefield evaluated over Ω [62], [63] with time delays defined in (9)

(10) $$g_{\text{Ω}}(t)=\iint_{\text{Ω}}p(r,t+\tau (r))dr.$$

In practical applications of USCT, the acoustic medium is spatially heterogeneous and analytic expressions for p(r,t) are generally not available. Simulation of the transmitted or received measurement signal therefore requires use of a numerical wavesolver method as described below.

## III. USCT Forward Model for Use With Elevation-Focused Transducers

In this section, a new forward model for ring-array USCT that incorporates the elevational focusing properties of the transducers is formulated. The forward model is presented first in a C-C form then in its D-D form. The adjoint model for use in FWI is also provided in a D-D form.

### A. C-C Forward Model Incorporating Focusing Effects

Here, a lens-focused transducer model will be combined with a wave propagation model to establish an overall forward model for lens-focused USCT. To accomplish this, the relationship between the source pulse $s_{i}(r,t)$ in (1) and the normal velocity $v_{⊥}(r,t)$ in the transducer model (7) is described and a new sampling operator and its adjoint are introduced.

For the rigid baffle transducer model employed in Section II, the forcing term $s_{i}(r,t)$ can be modeled as a mass source in (1) [64]. Specifically, the forcing term has the form

(11) $$s_{i}(r,t)=\chi_{i}(r)\frac{\partial }{\partial t}S_{m}(r,t)=2{\hat{\rho }}_{0}\chi_{i}(r)\frac{\partial }{\partial t}v_{⊥}(r,t)$$

where ${\hat{\rho }}_{0}$ is the ambient density, $S_{m}(r,t)=2\hat{\rho_{0}}v_{⊥}(r,t)$ is a mass source, $v_{⊥}(r,t)$ and $\chi_{i}(r)$ are the normal component of the velocity and the indicator function describing the support of the ith transducer as introduced above [64], [65].

By using (8), (11) can be reexpressed as

(12) $$s_{i}(r,t)=2{\hat{\rho }}_{0}\chi_{i}(r)\frac{\partial }{\partial t}\hat{v}\left(t+\tau_{i}(r)\right)$$

where $\tau_{i}(r)$ is the time delay function associated with the ith transducer. Equation (12) defines the excitation source in the lens-focused USCT forward model provided below.

This source can be further characterized in terms of a mapping operator ${\left({ℳ}_{i}^{\tau }\right)}^{†}:L^{2}([0,T])\mapsto L^{2}\left({\mathbb{R}}^{3}\times [0,T]\right)$. First, let $D_{\tau }:L^{2}([0,T])\mapsto L^{2}([0,T])$ denote a time shift operator that satisfies $\phi_{\tau }(t)=D_{\tau }\phi (t):=\phi (t+\tau )$, where ϕ(t) is a 1-D continuous signal. Additionally, let $x(t):=2{\hat{\rho }}_{0}\frac{\partial }{\partial t}\hat{v}(t)$. In terms of these quantities, the source and operator ${\left({ℳ}_{i}^{\tau }\right)}^{†}$ are related as

(13) $$s_{i}(r,t)={\left({ℳ}_{i}^{\tau }\right)}^{†}x(t):=\chi_{i}(r)D_{\tau_{i}(r)}x(t).$$

The measurement data $g_{ij}\left(t\right)$ produced by the ith source and measured by the jth transducer can be described by use of the sampling operator $\left({ℳ}_{j}^{\tau }\right)$ as

(14) $$g_{ij}(t)=\left({ℳ}_{j}^{\tau }\right)p_{i}(r,t):=\int_{{\mathbb{R}}^{3}}\chi_{j}(r)D_{\tau_{j}(r)}p_{i}(r,t)dr.$$

Finally, the idealized C-C forward model in (3) can be generalized to the case of elevational-focused USCT as

(15) $$g_{ij}(t)={ℳ}_{j}^{\tau }{ℋ}^{u(r)}s_{i}(r,t)\;\text{with}\;(s_{i}(r,t)={\left({ℳ}_{i}^{\tau }\right)}^{†}x(t).$$

### B. D-D Imaging Model Incorporating Focusing Effects

Here, a D-D version of (15) is provided. First, the discrete counterpart of the time delay operator $D_{\tau }$, denoted as $D_{\tau }\in {\mathbb{R}}^{L\times L}$, is defined as

(16) $$D_{\tau }\phi :=F^{-1}[\exp(-j2\pi k\tau )⊙(F\phi )]$$

where $\phi \in {\mathbb{R}}^{L}$ denotes a 1-D discrete temporal signal, F and $F^{-1}$ denote the 1-D discrete Fourier transform and its inverse, ⊙ represents element-wise multiplication, and k is a set temporal frequencies defined as

$$k=\{\begin{array}{ll} \frac{2\pi }{L\text{Δ}t}\left[-\frac{L-1}{2},-\frac{L-1}{2}+1,\ldots ,\frac{L-1}{2}\right], & \text{if}\;L\;\text{is odd}\\ \frac{2\pi }{L\text{Δ}t}\left[-\frac{L}{2},-\frac{L}{2}+1,\ldots ,\frac{L}{2}\right], & \text{if}\;L\;\text{is even}. \end{array}$$

As defined in Section II-A, L denotes the number of temporal samples of the signal that are spaced by Δt Note that (16) allows for arbitrary time delays to be implemented that are not necessarily exact multiples of the sampling interval Δt.

**Fig. 1.**
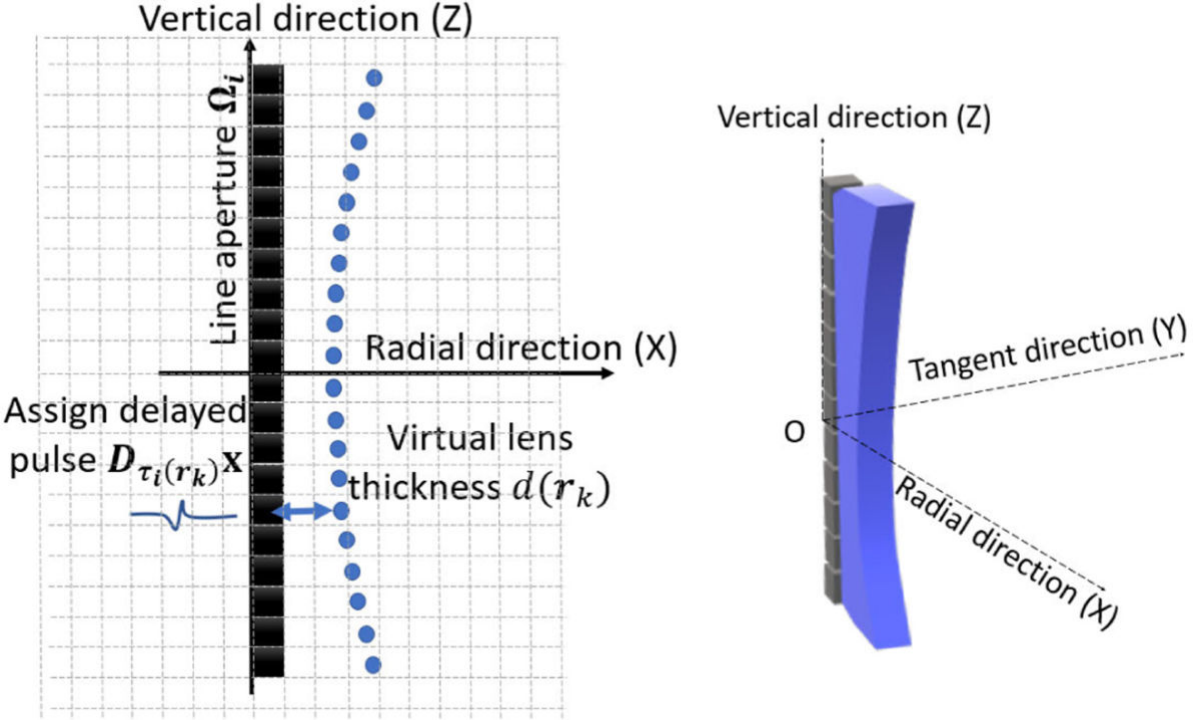
Schematic of a discretized focused transducer. The transducer aperture is approximately a line segment due to the high aspect ratio. The vertical, radial, and tangent directions are referred as the Z-, X-, and Y-axis, respectively. The radial direction is toward the center of the imaging system.

Next, the transducer aperture Ω is discretized to conform to the assumed computational grid. Due to the high aspect ratio of the transducers often employed in ring-array USCT [31], [32], the transducer width is neglected and the aperture is described approximately by a line that is parallel to the vertical axis and whose length corresponds to the height of the transducer. The line aperture is divided into N consecutive line segments, with the length of each segment corresponding to height of a voxel in the computational grid. An example of a lens-focused transducer and such segmented line aperture is depicted in Fig. 1. When the line aperture does not evenly bisect the voxels, a nearest-neighbor interpolation method is employed where each segment is assigned to the nearest grid point. Hereafter, the set $K_{{\text{Ω}}_{i}}\subset \{0,1,\ldots ,K-1\}$ will denote the collection of indices k corresponding to the grid points associated with the ith transducer aperture $\left(r_{k}\in {\text{Ω}}_{i}\right)$. The cardinality of $K_{{\text{Ω}}_{i}}$ is the number N of consecutive line segments that form the line aperture.

Next, as a prerequisite for establishing the D-D forward model, discrete versions of (13) and (14) are established. First, a sampling matrix $M_{i}^{\tau }$ corresponding to the jth receiver is introduced as the discrete counterpart of ${ℳ}_{j}^{\tau }$. It is defined as $M_{j}^{\tau }:=\sum_{k\in K_{{\text{Ω}}_{j}}}{\tilde{M}}^{k}⊗D_{\tau_{j}\left(r_{k}\right)}$, where $\tau_{j}(r_{k})$ is the time delay of the jth transducer at a position $r_{k}$, and ${\tilde{M}}^{k}\in {\mathbb{R}}^{K\times 1}$ is an indicator vector. Specifically, ${\left[{\tilde{M}}^{k}\right]}_{i}=\delta_{ik}$ such that the kth element is equal to 1 and other elements are all zero, where $\delta_{ik}$ is a Kronecker delta function. Similarly, the mapping matrix for the ith transmitter, which is the transpose of the sampling matrix, is defined as ${\left(M_{i}^{\tau }\right)}^{⊤}:=\sum_{k\in K_{{\text{Ω}}_{i}}}{\left({\tilde{M}}^{k}\right)}^{⊤}⊗D_{\tau_{i}\left(r_{k}\right)}$. Note that the time shift operator is self-adjoint and the Kronecker product is commutative.

To establish the discrete counterpart of (13), the spatial-temporal source term is specified by use of the mapping matrix ${\left(M_{i}^{\tau }\right)}^{⊤}$. Specifically, for the ith transmitter, the source term is defined as

(17) $${\left[s_{i}\right]}_{kL+l}={\left[{\left(M_{i}^{\tau }\right)}^{⊤}x\right]}_{kL+l}:=\{\begin{array}{ll} {\left[D_{\tau_{i}\left(r_{k}\right)}x\right]}_{l}, & \forall k\in K_{{\text{Ω}}_{i}}\\ 0, & \forall k\notin K_{{\text{Ω}}_{i}}. \end{array}$$

For k=0,1,...,K−1 and l=0,1,...,L−1, where $x\in {\mathbb{R}}^{L}$ is a 1-D discretized signal temporally sampled from the continuous velocity source as ${\left[x\right]}_{l}:=2{\hat{\rho }}_{0}\frac{\partial }{\partial t}\hat{v}\left(t_{l}\right)$. In (17), the source $s_{i}$ can be interpreted as a superposition of temporally delayed contributions produced by the N line segments belonging to the ith transducer ${\text{Ω}}_{i}$.

To establish the discrete counterpart of (14), the pressure data recorded by receivers is specified by use of the sampling matrix $M_{j}^{\tau }$. Specifically, the discrete pressure data $g_{ij}$ that are produced by the ith source and recorded at the jth transducer are described as

(18) $$g_{ij}=M_{j}^{\tau }p_{i}:=\sum_{k\in K_{{\text{Ω}}_{j}}}D_{\tau_{j}\left(r_{k}\right)}p_{i}\left(r_{k}\right)$$

where $p_{i}\left(r_{k}\right)$ denotes 1-D temporal discrete pressure data at the grid point $r_{k}$ generated by source $s_{i}$, and the summation is over the jth receiver aperture ${\text{Ω}}_{j}$. In (18), the received data are formed as a superposition of discrete pressure signals corresponding to different locations on the transducer aperture, each with appropriately defined time shifts.

Finally, a D-D version of the C-C forward model in (15) is obtained as

(19) $$g_{i}=M^{\tau }H^{\text{u}}s_{i}\;\text{with}\;s_{i}={\left(M_{i}^{\tau }\right)}^{⊤}x$$

where $M^{\tau }:=\left[M_{0}^{\tau_{0}};M_{1}^{\tau_{1}};\ldots ;M_{M-1}^{\tau_{M-1}}\right]\in {\mathbb{R}}^{ML\times KL}$. This equation represents a generalization of the idealized D-D forward model in (5) to the case of elevational-focused USCT.

### C. Formulation of the Adjoint Equation

Similar to the formulation reviewed in Section II-B and reusing the same notation, the reconstruction problem in 3-D ring-array based USCT can be defined as

(20) $$\hat{u}=\arg\underset{u}{\min}E_{w}\frac{1}{2}{\left\|\underline{g^{w}}-M^{\tau }H^{u}s^{w}\right\|}_{2}^{2}+\text{λ}R(u).$$

To enable reconstruction of the SOS distribution, the gradient of data fidelity term in (20) with respect to c, denoted by $\nabla_{c}J^{w}$, can be computed via an adjoint state method [66], [67]. The discretized expression for the gradient is given by [13], [18], and [68]

(21) $${\left[\nabla_{c}J^{w}\right]}_{k}=\frac{1}{{\left[c\right]}_{k}^{3}}\sum_{l=1}^{L-2}{\left[q^{w}\right]}_{kL+(L-l)}\;\;\times \frac{\left({\left[p^{\text{w}}\right]}_{kL+l-1}-2{\left[p^{\text{w}}\right]}_{kL+l}+{\left[p^{\text{w}}\right]}_{kL+l+1}\right)}{\text{Δ}t}$$

where k, l are the indices of grid points and time steps, respectively. The quantity $p^{w}\in {\mathbb{R}}^{KL}$ denotes the wavefield data generated by the encoded source as $p^{\text{w}}=H^{\text{u}}s^{\text{w}}$ and $q^{w}\in {\mathbb{R}}^{KL}$ denotes the adjoint wavefield data that are computed as

(22) $$\;q^{\text{w}}=H^{\text{u}}{\left(M^{\tau }\right)}^{⊤}\eta^{w};\;\text{with}\;{\left[\eta^{w}\right]}_{jL+l}:={\left[M^{\tau }H^{u}s^{w}-\underline{g^{w}}\right]}_{jL+(L-l)}.$$

Here, $\eta^{\text{w}}\in {\mathbb{R}}^{ML}$ is the time-reversed data residual and j is the index of the transducer.

Equation (21) holds when nonlossy media is considered. In attenuating media, (21) is an approximate expression for the gradient with respect to the SOS, as additional terms coming from AA modeling are neglected [68].

## IV. Validation and Characterization of the Imaging Models

In this section, the proposed forward model is validated and characterized. First, the forward model is validated by use of a semianalytical approximation of the Rayleigh–Sommerfield diffraction integral solution for the case of a lossless homogeneous medium. Next, a sensitivity map is computed to describe the region of a 3-D object that is effectively “seen” by a specified focused emitter–receiver pair in a ring-array system.

**Fig. 2.**
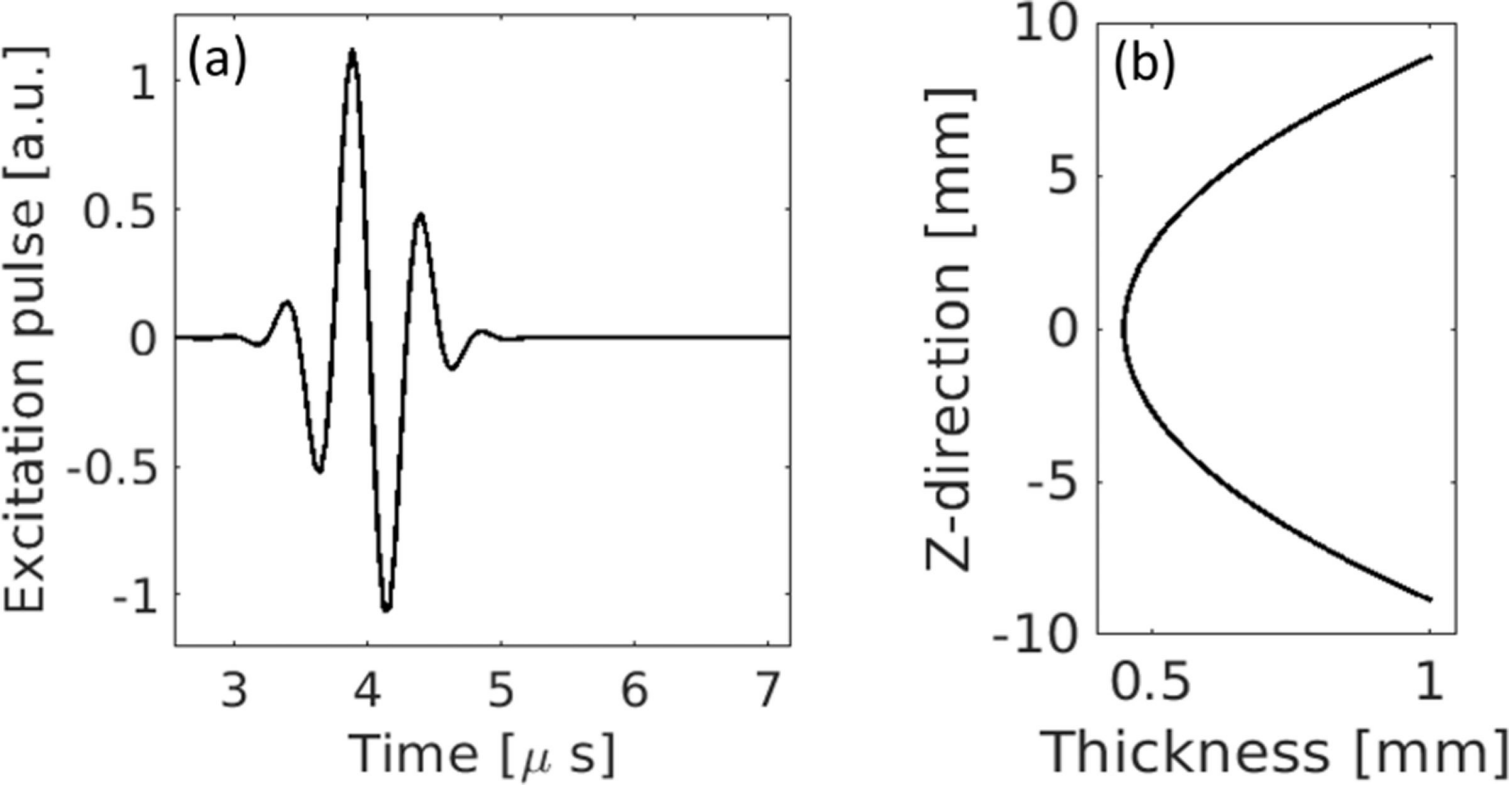
(a) Excitation source pulse employed in the validation studies. (b) Profile of the lens thickness $d(z)=(\text{a}/2)z^{2}+d_{0}$ with curvature $a=0.014\;{\text{mm}}^{-1}$ and offset $d_{0}=0.45\;\text{mm}$. Note that plot is not to scale and the horizontal axis representing the thickness of the lens has been stretched for visualization purposes.

In the following studies, all emitters and receivers were specified using the same elevation-focused transducer model. Specifically, a line transducer with a height of 18 mm and a concave lens with a parabolic curvature was considered. The SOS in the lens was set as $c_{\text{lens}}=4500\;\text{m/s}$. The shape of the excitation source is shown in Fig. 2(a), and the profile of the lens is shown in Fig. 2(b). The source pulse had a central frequency of 2 MHz and a maximum frequency of 3.5 MHz. Additional information regarding the employed source pulse, acoustic lens curvature, and associated time delays can be found elsewhere [37].

### A. Validation of the Forward Model

In the first validation study, the pressure wavefield produced by use of the numerical transducer model and measured by use of an ideal point transducer was compared to the reference solution described in (7)–(9). This is equivalent to validating the source $s_{i}={\left(M_{i}^{\tau }\right)}^{⊤}x$ and operator $H^{\text{u}}s_{i}$. The reference solution was computed in a semianalytical way, where the integrals were computed numerically by the distributed point source method [69]. The action of the operator $H^{\text{u}}$ was implemented by use the k-space pseudospectral method [65], [70]. The computational domain was a uniform 3-D Cartesian grid with 1280 × 1280 × 144 cubic voxels. Each voxel had a dimension of 0.2 mm. Accordingly, the transducer was divided into a 1-D array of 90 voxels, corresponding to a height of 18 mm. The discretized source term $s_{i}$ with varying time delays was assigned to the 90 voxels as multiple mass sources. To ensure accurate simulation using pseudospectral methods, the spatial discretization of the pressure wavefield was set to 3 points per wavelength [70]. To ensure numerical stability, the Courant–Friedrichs–Lewy (CFL) number was set to 0.3, which yielded a time step size of 0.04 *μ*s. The medium was assumed to be lossless and homogeneous with a SOS of 1500 m/s and a density of 1000 kg/m^3^.

**Fig. 3.**
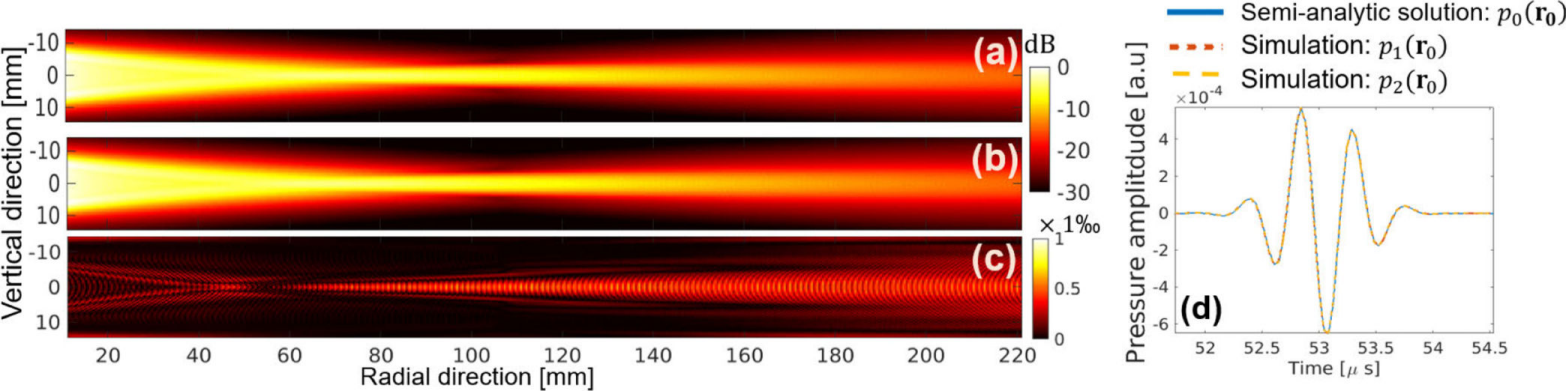
Maximum pressure amplitude maps in a transverse plane are displayed, using (a) numerical transducer model and (b) semianalytical reference solution. These maps are presented on a decibel scale and computed using the formula $20\log_{10}\left(\max_{t}\left|p_{i}\right|/\max_{r,t}\left|p_{i}\right|\right)\;(i=1,2)$, where $p_{0}=p_{0}(r,t)$ and $p_{1}=p_{1}(r,t)$ represent the pressure fields computed by the semianalytic solution and the proposed method, respectively. The relative difference map of the two solutions is shown in (c). The map is normalized by the maximum value of the on-axis (z=0mm) semianalytic solution $\left(\left|\max_{t}p_{0}-\max_{t}p_{1}\right|/\left|\max_{t}p_{0}\left(r_{1},t\right)\right|\right)$, where $r_{1}=(x,0,0)$). The focal region is situated at a distance ranging from 60 to 140 mm from the transducer. (d) Validation of the operator $H^{\text{u}}s_{i}(p_{0}\left(r_{0}\right)\;\text{vs}\;p_{1}\left(r_{0}\right))$ and the overall forward operator $M_{j}^{\tau }{\text{H}}^{\text{u}}s_{i}$ is conducted using acoustic reciprocity $(p_{0}\left(r_{0}\right)\;\text{vs}\;p_{2}\left({\text{r}}_{0}\right))$. Here, $p_{0}\left(r_{0}\right)$ and $p_{1}\left(r_{0}\right)$ denote the pressure measured at a specific location $r_{0}(x=71\;\text{mm},y=1\;\text{mm},z=0.1\;\text{mm})$, while $p_{2}\left(r_{0}\right)$ represents the response of the lens-focused transducer when a point-like source is at location $r_{0}$. The source pulse depicted in Fig. 2(a) was employed to compute all pressure fields $p_{0},p_{1}$, and $p_{2}$.

Fig. 3(a) and (b) shows spatial maps of the recorded maximum pressure amplitude in a lateral plane, corresponding to the numerical model $\left(p_{1}\right)$ and reference method $\left(p_{0}\right)$. Also, the spatial relative difference map of the two solutions is shown in Fig. 3(c), where relative error is less than 0.1% over all the spatial domain. Plots of the time-varying pressure signal at a fixed measurement location $r_{0}$ are compared in Fig. 3(d). The pressure signals produced by use of the numerical $\left(p_{1}(r_{0})\right)$ and reference methods $\left(p_{0}(r_{0})\right)$ are nearly identical. These results serve as a validation of the operator $H^{\text{u}}s_{i}$, which is a special case of the forward model when an idealized point receiver is assumed.

To validate the overall forward operator $M_{j}^{\tau }H^{\text{u}}s_{i}$ for use with lens-focused receiving transducers, a numerical experiment was designed in which the validated operator $H^{\text{u}}s_{i}$ and the principle of acoustic reciprocity were involved. Traditionally, the reciprocity principle is stated in terms of a monopole point source and a point receiver; however, the same principle also holds for transducers with an extended aperture and lens curvature [71]. Here, the validation study exploited the fact that the pressure signal measured by the lens-focused transducer in response to a point source is equivalent to the pressure signal produced by the lens-focused transducer and observed at the point source location, assuming the same excitation source pulse is used. A simulation was performed that employed an idealized point transducer emitter located at $r_{0}$. The focused emitter used for the validation of $H^{\text{u}}s_{i}$ was now employed as a receiver to record the signal produced by the point emitter, which is denoted as $p_{2}(r_{0})$ in Fig. 3(d). The close agreement between $p_{0}(r_{0})$ and $p_{2}(r_{0})$ in Fig. 3(d) serves as a validation of the overall transducer model.

### B. Characterization via the Sensitivity Map

**Fig. 4.**
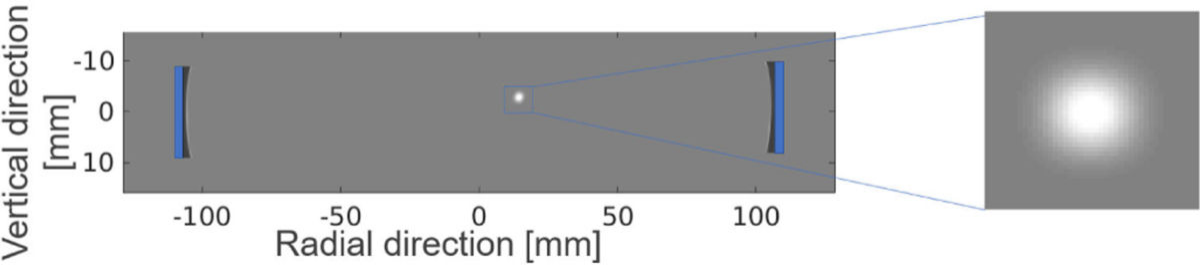
Schematic of the transducer locations and the point target that was employed in the sensitivity map calculation. The transducer emitter–receiver pair is shown as blue rectangles. The point target: a blurred object with a peak SOS value of 1570 m/s.

To characterize the combined focusing effect of a given emitter–receiver transducer pair, a sensitivity map was computed. This served to visualize the region of the 3-D object that can significantly influence the data recorded by that pair. The sensitivity map S(r) is defined as

(23) $$S(r)\equiv \frac{\int {\left(p_{r}(t)-p_{0}(t)\right)}^{2}dt}{\int {\left(p_{0}(t)\right)}^{2}dt}$$

where $p_{0}\left(t\right)$ is the recorded pressure signal corresponding to the homogeneous medium and $p_{r}\left(t\right)$ is the recorded pressure signal when the point target is inserted at a location **r**. As such, the value at each location in the sensitivity map describes how the measured pressure data corresponding to a homogeneous medium would be perturbed by a point target at that location. In this study, the homogeneous medium was defined by the following acoustic parameters: $c_{0}=1500\;\text{m/s}$, $\rho_{0}=\;1000\;{\text{kg/m}}^{3}$, and α=0. The inserted point target was modeled as a 3-D Gaussian perturbation in the SOS. After the insertion of a point target at location r, the SOS distribution was specified as $c_{r}\left(r^{\prime }\right)=c_{0}+c_{1}e^{-\left({\left(r^{\prime }-r\right)}^{2}/2\sigma^{2}\right)}$, where $c_{1}=70\;\text{m/s}$ and σ=0.8mm. Fig. 4 shows the relative location of a diametrically opposed emitter–receiver separated by a distance of 220 mm and an illustration of the inserted point target. The sensitivity map was computed by varying r within the field of view.

**Fig. 5.**
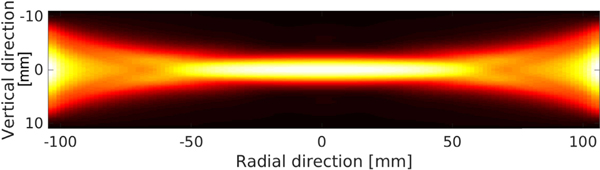
Transverse sensitivity map computed by use of (23) for a diametrically opposed emitter–receiver pair. This map depicts the elevation focusing achieved for the emitter–receiver pair.

Fig. 5 shows the computed sensitivity map. Point targets were uniformly distributed between −10.8 and 10.8 mm in the vertical direction with a spatial sampling interval of 0.4 mm, and between −104 and 104 mm in the radial direction with a spatial sampling interval of 3.2 mm. The map depicts a thin region (about 6 mm) of high sensitivity to acoustic heterogeneities near the elevation focus.

By enabling visualization of the effective focusing effect achieved by a given emitter–receiver pair, the sensitivity map can provide insights into the vertical resolution of ring-array USCT systems. Additionally, it can guide the choice of parameters used in the reconstruction method, such as the height of the required computational domain. If measurements acquired at multiple ring-array locations are to be utilized together for image reconstruction, the sensitivity map can also guide the design of the step size by which ring-array should be vertically translated during data acquisition.

## V. Case Study

To demonstrate the utility of the proposed forward models for ring-array-based USCT, a virtual imaging study that employed realistic 3-D NBPs was conducted. This study sought to understand whether 3-D image reconstruction of a slab-shaped volume could yield improved image quality as compared to the conventional 2-D SBS approach. The impact of transducer modeling errors on reconstructed 3-D images was also investigated.

### A. Virtual Imaging System

A virtual ring-array-based USCT system with elevational focusing prescribed by the proposed focused transducer model was assumed. The imaging system comprised 128 elevation-focused emitters and 1024 elevation-focused receivers that were evenly arranged in a ring array of a radius of 110 mm [32], [34]. The transducer parameters assumed in Section IV were employed. Specifically, a lens with parabolic curvature $a=0.014\;{\text{mm}}^{-1}$ [see Fig. 2(b)] and SOS $c_{\text{lens}}=4500\;\text{m/s}$ was assumed. During data acquisition, measurement data at three different ring-array locations, referred to as ring −1, 0, +1, were acquired by translating the ring-array vertically and stopping at three equispaced locations. The distance between two consecutive scanned imaging planes was 1.8 mm, corresponding to one-tenth of the transducer height. In the subsequent reconstruction studies, the multiring data were only utilized to generate a good initial guess for 3-D studies. Only single-ring data from ring 0 were utilized in the final reconstruction.

**Fig. 6.**
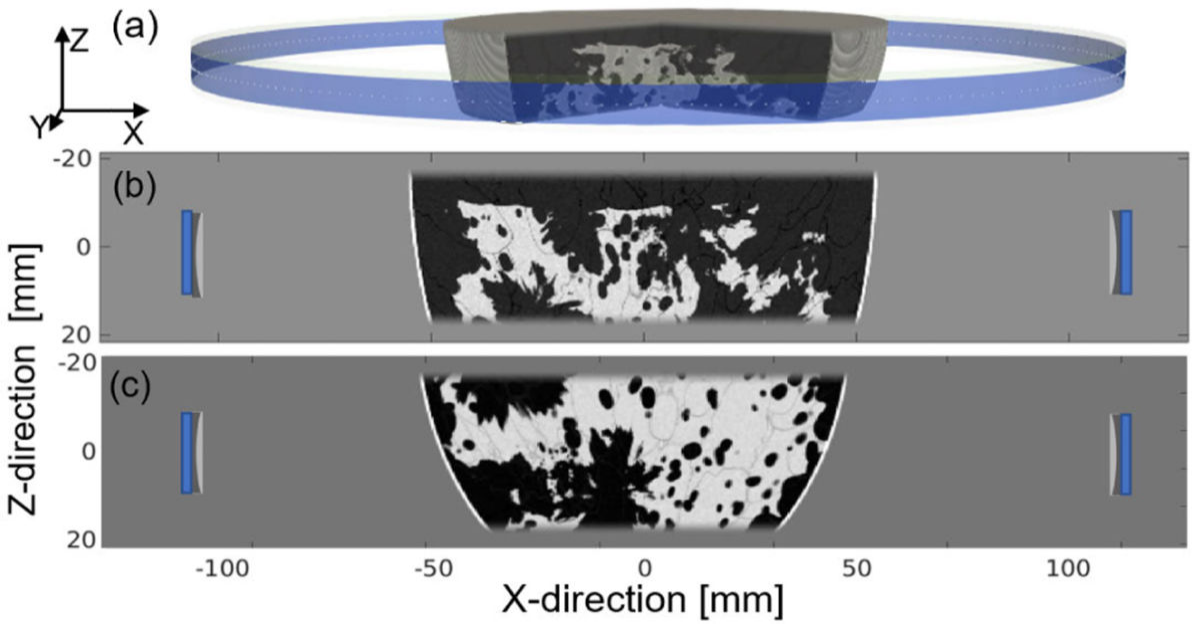
Schematic of the virtual imaging system used in the case study. (a) Three-dimensional rendering of the ring-array USCT system and the object. (b) Transverse plane view of the thin-slab SOS breast phantom of breast type B. (c) Thin-slab SOS phantom of breast type C.

Thin slabs extracted from two 3-D anatomically realistic NBPs [72] corresponding to a BI-RADS breast density category B (scattered areas of fibroglandular density) and C (heterogeneously dense) were virtually imaged. Heterogeneous SOS, AA, and density distributions were considered, with realistic values of the tissue properties assigned [72]. Fig. 6(b) and (c) shows cross-sectional maps of the SOS distribution of the type B and type C NBPs in the transverse plane, respectively. Note that the field of view for the breast type C was chosen near the nipple, resulting in a pronounced vertical curvature.

Further implementation details regarding the wave simulations and measurements data acquisitions are presented in Appendix C.

### B. Image Reconstruction Studies

The goal of the studies was to investigate the robustness of 3-D FWI to transducer modeling errors, as it can be challenging to accurately represent the focusing properties of actual transducers due to their complex design, so that a small modeling mismatch may exist between the actual transducer and the computational model. To this end, slab-shaped SOS volumes were reconstructed using 3-D FWI and the proposed forward model, with the exact numerical transducer model used in the forward data generation. An additional 3-D reconstruction study was conducted using a transducer model with distorted lens curvature. The transducer model overestimated the curvature of the lens by 10% $\left(a=0.0154\;{\text{mm}}^{-1}\right)$, which can lead to inaccurate time delays and focusing effects. As a reference, SOS images were reconstructed by 2-D FWI using measurement data acquired at ring 0. The transducers were modeled as point-like sources/receivers in the imaging plane. To compare the estimated SOS from the 2-D and 3-D models, the 2-D FWI-reconstructed image was compared to 2-D slices of the 3-D FWI-reconstructed images at the imaging plane of ring 0.

In these reconstruction studies, a two-step coarse-to-fine grid method for time-domain FWI was used to address the issue of constructing a good initial SOS model to avoid cycle-skipping and reduce the computational burden. In the first step, a homogeneous SOS medium $\left(c_{0}=1500\;\text{m/s}\right)$ was used as the starting model and FWI was performed on a coarser grid using lower frequency measurements, to estimate a good initial guess for finer grid reconstruction. In the second step, the estimated SOS map from the first step was refined on a finer grid using higher frequency data. Specifically, in all 3-D reconstructions, initial guesses were obtained by combining three coarse grid SOS estimates using measurements acquired from rings −1, 0, and +1. This approach, which uses multiring data, allows for a more accurate SOS representation of the object at different elevation positions, particularly in the high-sensitivity region shown in Fig. 5. However, only measurements at ring 0 were used in the final finer grid FWI. In all reconstructions, a heuristic approach to compensate for the heterogeneity of AA and density media was used. Specifically, a two-region piecewise constant model of AA/density media was used that assumes that the breast boundary is known (reflectivity imaging could be possibly used to estimate it) and assigns a constant AA/density value to the background and another to the breast region, which corresponds to a weighted average of the mean values of AA/density in fatty and glandular tissues [72]. The heuristic approach is designed as a fair approach for comparing 2-D and 3-D methods. The accurate reconstruction of SOS with AA/density mismatches and the impact of such mismatches are not the primary focus of this article [73]. Additionally, measurements were corrupted with Gaussian-independent and identically distributed (i.i.d.) noise. The implementation details of the two-step reconstruction method, the approach of the initial guess formation from three coarse grid reconstructions, the use of perturbed time delays, and the noise model are provided in Appendix C.

### C. Results

**Fig. 7.**
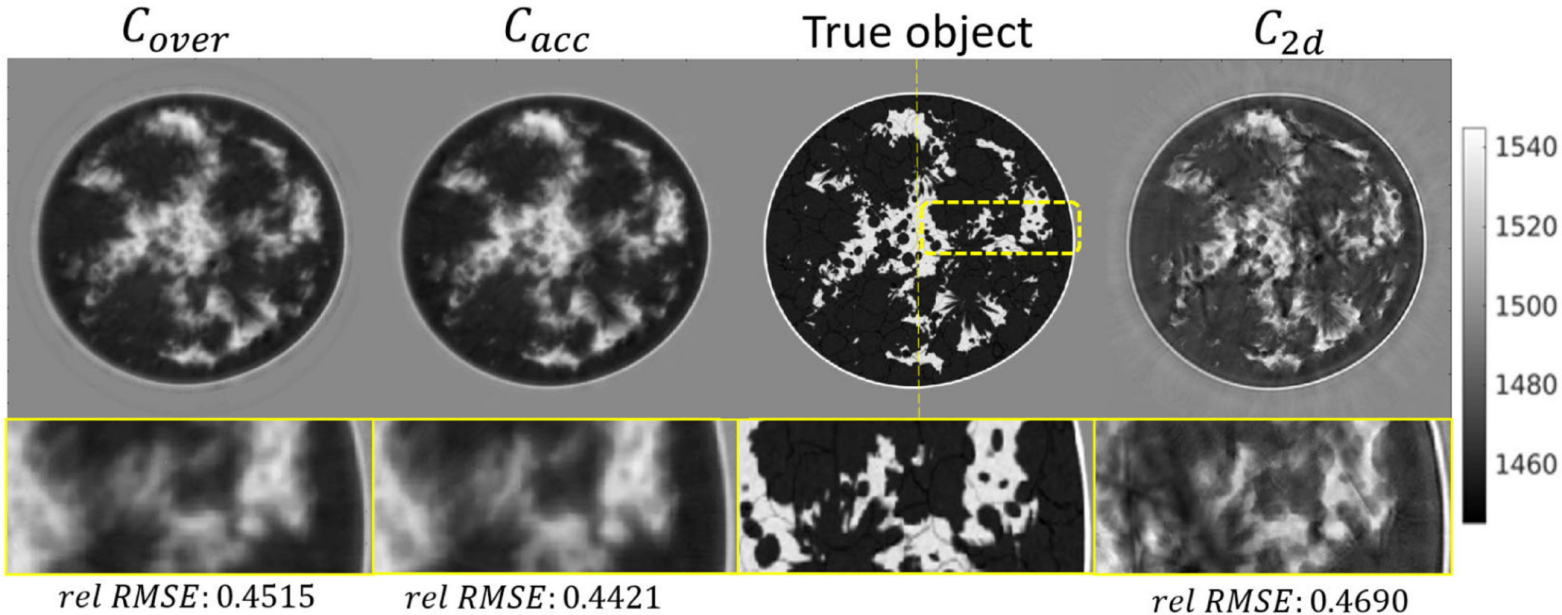
Breast type B: Cross-section view of reconstructed SOS distribution by 3-D and 2-D time-domain FWI at the imaging plane of ring 0. The colorbar is displayed in units of m/s. The rel-RMSE evaluates the relative error between the slice at imaging plane and the true 2-D slice and $\text{rel-RMSE}(c)=\left({\left\|c-c_{\text{true}}\right\|}_{2}/{\left\|c_{0}-c_{\text{true}}\right\|}_{2}\right)$. The quantities $C_{\text{over}}$, $C_{\text{acc}}$ denote the object reconstructed by 3-D FWI using an overestimated and an accurate transducer model, respectively; $C_{2d}$ denotes the SOS image estimated by 2-D FWI. The yellow bounding box in True object indicates the selected region shown at the bottom row images. The vertical yellow dashed line indicates the location of the line profiles presented in Fig. 9.

**Fig. 8.**
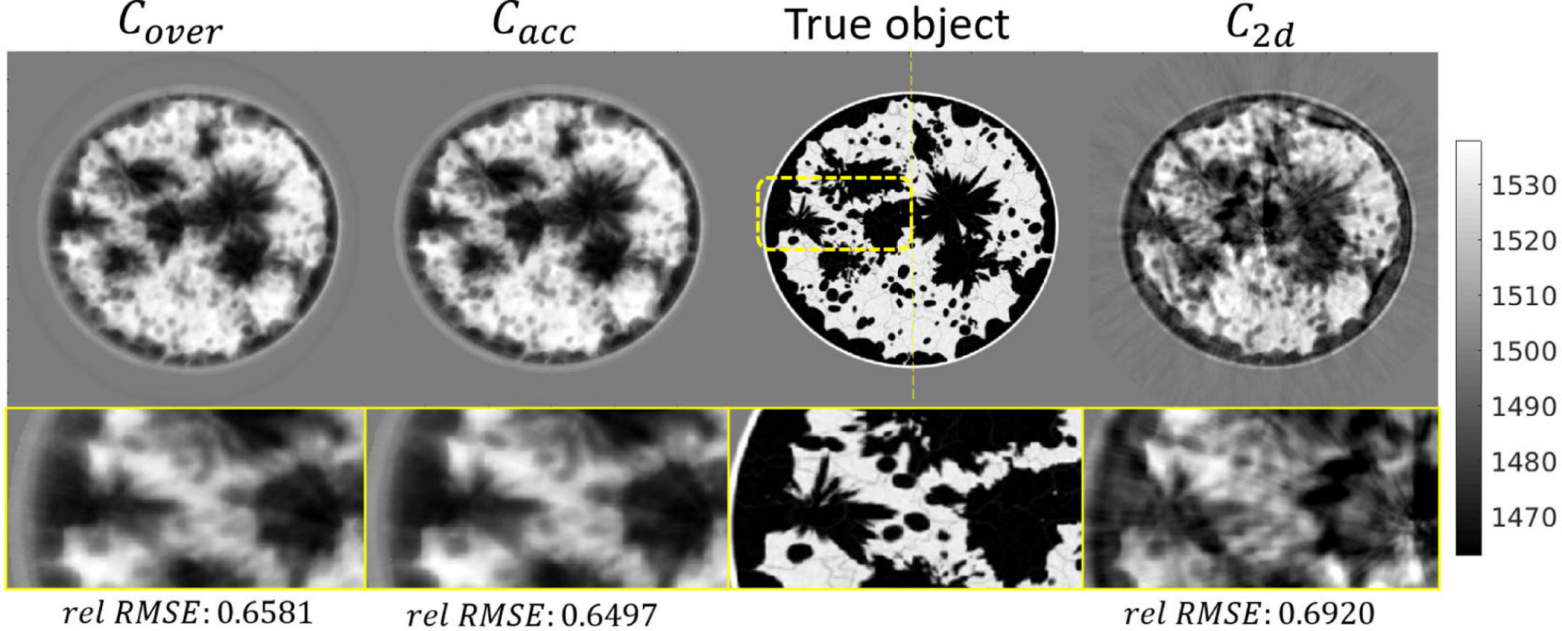
Breast type C: Cross-section view of the reconstructed SOS distribution by 3-D and 2-D time-domain FWI at the imaging plane of ring 0. The colorbar is displayed in units of m/s.

**Fig. 9.**
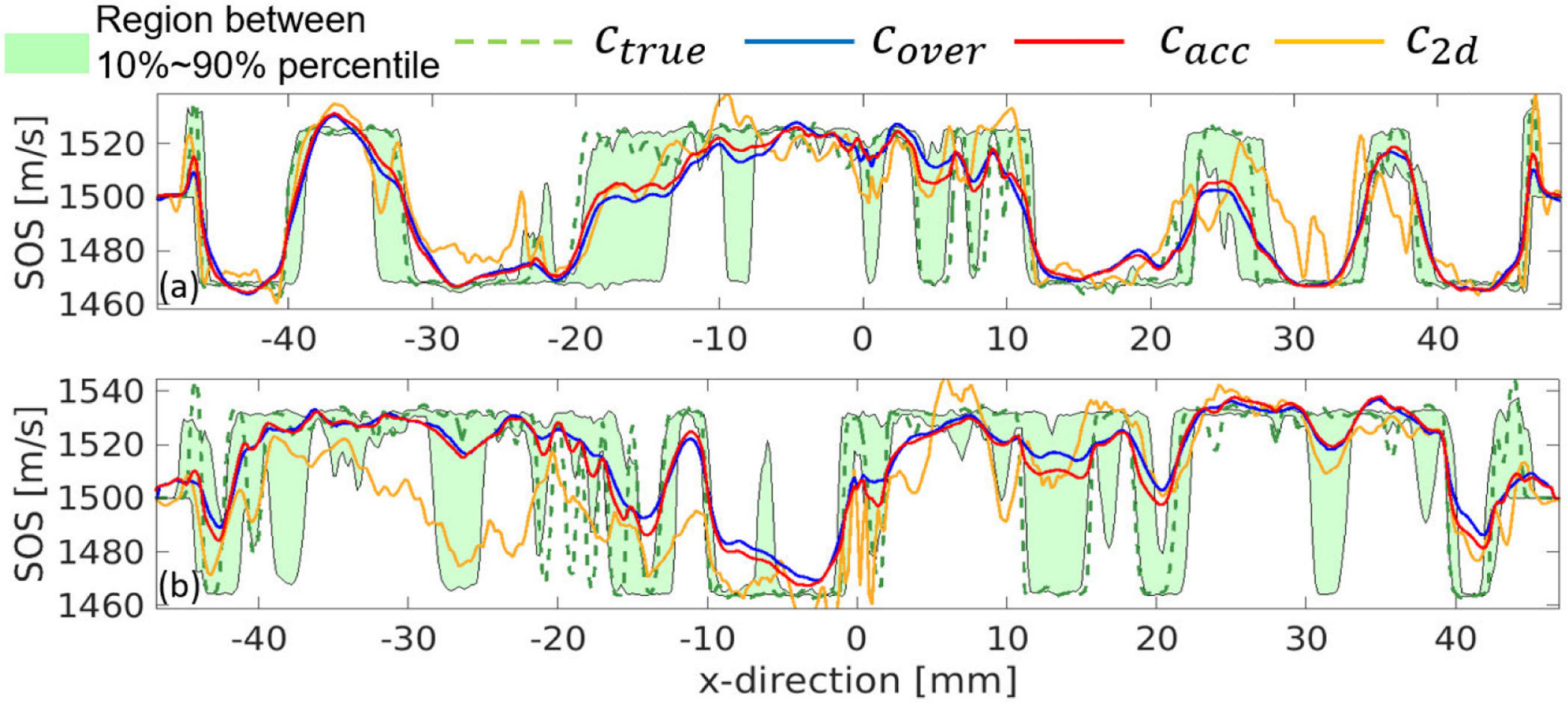
Line profiles at y=0 mm for comparing the estimated SOS using different transducer models. (a) Breast phantom type B and (b) type C.

The reconstructed SOS maps at ring 0 are shown in Fig. 7 for breast phantom type B, and Fig. 8 for breast phantom type C. Line profiles corresponding to the presented 2-D SOS maps (at the thin yellow lines) are shown in Fig. 9. The green shaded region indicates the vertical variance of true SOS in a high-sensitivity region (from −4 to 4 mm), where the upper and bottom boundaries are the 90% percentile and 10% percentile of the SOS distribution of each z-column. The shaded region reveals the large SOS heterogeneity of the employed phantoms along the vertical direction.

## VI. Discussion

The results of the case study show that modeling wave propagation in 3-D and accounting for elevation focusing can overcome the limitation of the 2-D approach and enable high-resolution estimates of SOS in the imaging plane, even when data from only a single elevation of the ring-array are used. Particularly, the 2-D reconstruction presents significant artifacts and incorrect tissue structures due to the out-of-plane scattering and 2-D/3-D model mismatch. Notably, these image artifacts are similar to those observed in USCT images obtained from actual experimental data by the 2-D method [10]. The object estimates produced by 3-D FWI have a more accurate estimation of tissue structures, SOS values, and lower artifacts level, even when the transducer curvature (and thus its focusing properties) are not known exactly. More importantly, as shown in Fig. 9, the profiles of 3-D FWI results mostly reside within the shaded region, whereas the profiles of 2-D FWI do not. This shows that 3-D FWI results can provide SOS estimates that, despite the limited spatial resolution in the vertical direction, are consistent with SOS values of the 3-D object within the high-sensitivity region of the ring-array. The results demonstrated that such a 3-D reconstruction approach can improve the accuracy of SOS reconstruction, and it is robust with respect to uncertainty in the transducer model parameters.

However, mismatches clearly exist between the true object and the object estimated by 3-D FWI. Also, the 3-D FWI results appear smoother compared to the 2-D FWI. These are expected since only single-ring measurements were used in the finer grid reconstruction, which results in an underdetermined 3-D reconstruction problem. This is the cause of the limited spatial resolution of the images. On the other hand, the single-ring measurement data are sufficient for 2-D image reconstruction; however, substantial modeling errors arise by applying a 2-D imaging model to reconstruct data generated via 3-D wave propagation physics and lens focusing. As such, the images reconstructed by use of the 2-D method appear sharp but contain conspicuous artifacts due to the modeling error involved. This motivates the development of new algorithms incorporating USCT data corresponding to multiposition of the ring-array to further improve the spatial resolution of 3-D reconstruction [74].

The proposed model possesses a few limitations. First, the in-plane focusing (directivity) of the transducers is not modeled due to the high aspect ratio of the transducers employed in ring-array USCT. Additional discussions about modeling the directivity in ring-array USCT, especially when the in-plane aperture is not significantly larger than the pixel size, can be found in [40] and [75]. Second, the transducer model is Cartesian grid-based and requires the use of nearest-neighbor interpolation when the transducer location does not coincide with the voxel size. Non-Cartesian grids can also be explored as potential alternatives. One such approach is the isogeometric finite element method [76], which allows for an unstructured grid that can conform to the curved shape of the transducer [77], [78]. Nevertheless, it has challenges including computational cost and implementation difficulties, particularly when it comes to efficient hardware parallelizations. To reduce the modeling errors due to the irregular spacing in the non-Cartesian grid inside the transducer, one potential solution is applying varying weights scaling factors to different grid points. Third, the effects of the electrical impulse response (EIR) are not considered. However, the EIR can be readily incorporated as described elsewhere [63]. Fourth, in the employed lens-focused transducer model, the attenuation of the lens and the acoustic impedance mismatches at the lens surface are not taken into account. Related discussions are presented in Appendix B.

It is also worth noting that, in practical applications, lens parameters should be carefully designed for the desired focusing performance. An important consideration is the trade-off between the desired focusing performance and the loss induced by the lens (due to AA and acoustic impedance changes as described in Appendix B) as this could reduce the signal-to-noise ratio (SNR). In addition, the SOS value and curvature profile of the lens in simulation studies may not be optimal. Nevertheless, these are parameters of the proposed transducer model that can be adjusted as needed.

However, accurate transducer modeling for real experimental data remains a challenging task. To reduce transducer modeling errors for practical use, one approach involves calibration, which treats the excitation source pulse, transducer aperture, and time delays as a surrogate model. Further discussions on this topic can be found in [30] and [79].

Finally, in this study, only in-plane data acquisitions were employed to emulate the existing ring-array USCT system. However, the proposed model can be extended for more flexible cases. For instance, it can be adapted to model the case where receivers are positioned at different heights or where a vertically rotated ring-array configuration is utilized. Thus, the proposed model can be also used to explore new system geometries that could potentially offer improved vertical solutions.

## VII. Conclusion

In this study, a 3-D forward model on a regular Cartesian grid accounting for the elevation focusing effects in ring-array USCT systems was presented to enable accurate ultrasound simulation of the USCT data acquisition process. The focusing effects are modeled by decomposing each transducer into a discrete collection of source/receiver patches and applying a spatially varying time delay to the ultrasound pulse (emitter mode) and recorded signal (receiver mode). The proposed numerical transducer model was quantitatively validated by the use of a semianalytical approximation of the Rayleigh–Sommerfeld integral solution and of the acoustic reciprocity principle. The case study demonstrated that, by accounting for transducers’ focusing properties and 3-D wave propagation effects, the proposed 3-D imaging model can lead to more accurate estimates of SOS in the imaging plane and fewer artifacts compared to estimates obtained using a 2-D wave propagation model, such as that used in the SBS reconstruction approach. Furthermore, the case study demonstrated the robustness of 3-D FWI-based image reconstruction using the proposed imaging model with respect to uncertainty in the focusing properties of the transducer, which may be difficult to accurately characterize experimentally.

This work is a key step toward enabling improved volumetric image reconstruction in elevation-focused ring-array USCT. It also provides a foundation for conducting 3-D image reconstruction studies that employ data acquired at multiple ring-array positions.

## Figures and Tables

**Fig. 10.**
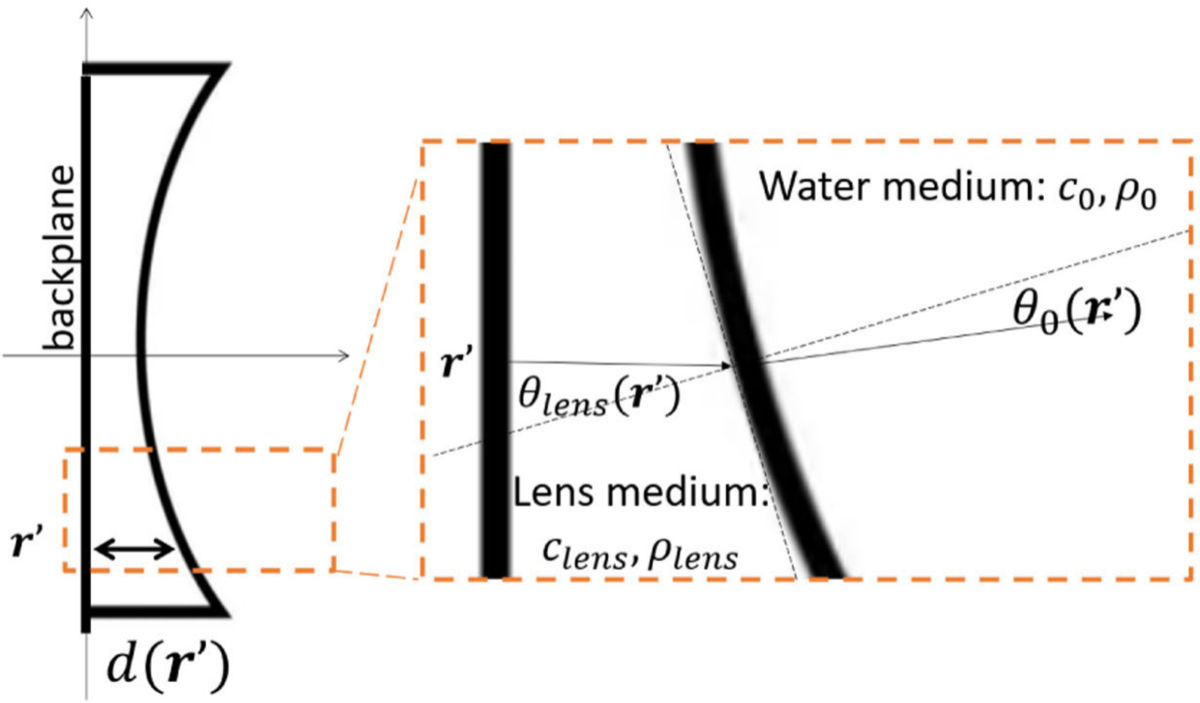
Illustration of lens thickness $d\left(r^{\prime }\right)$ and incident angle $\theta_{\text{lens}}$ and refraction angle $\theta_{0}$.

**Fig. 11.**
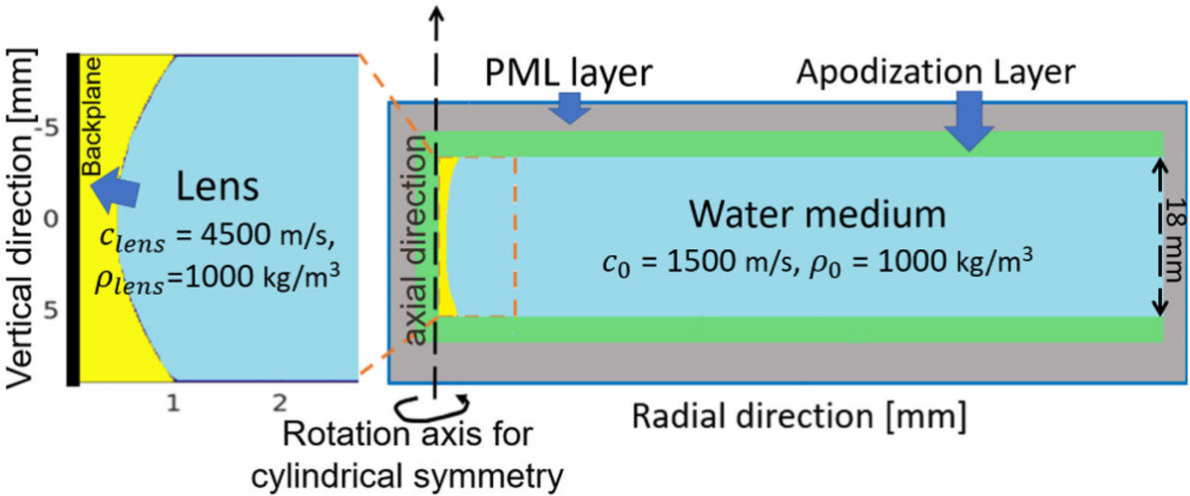
Illustration of a 2-D axisymmetric wave simulation medium. Here, the impedance of the lens is three times greater than the impedance of the water medium.

**Fig. 12.**
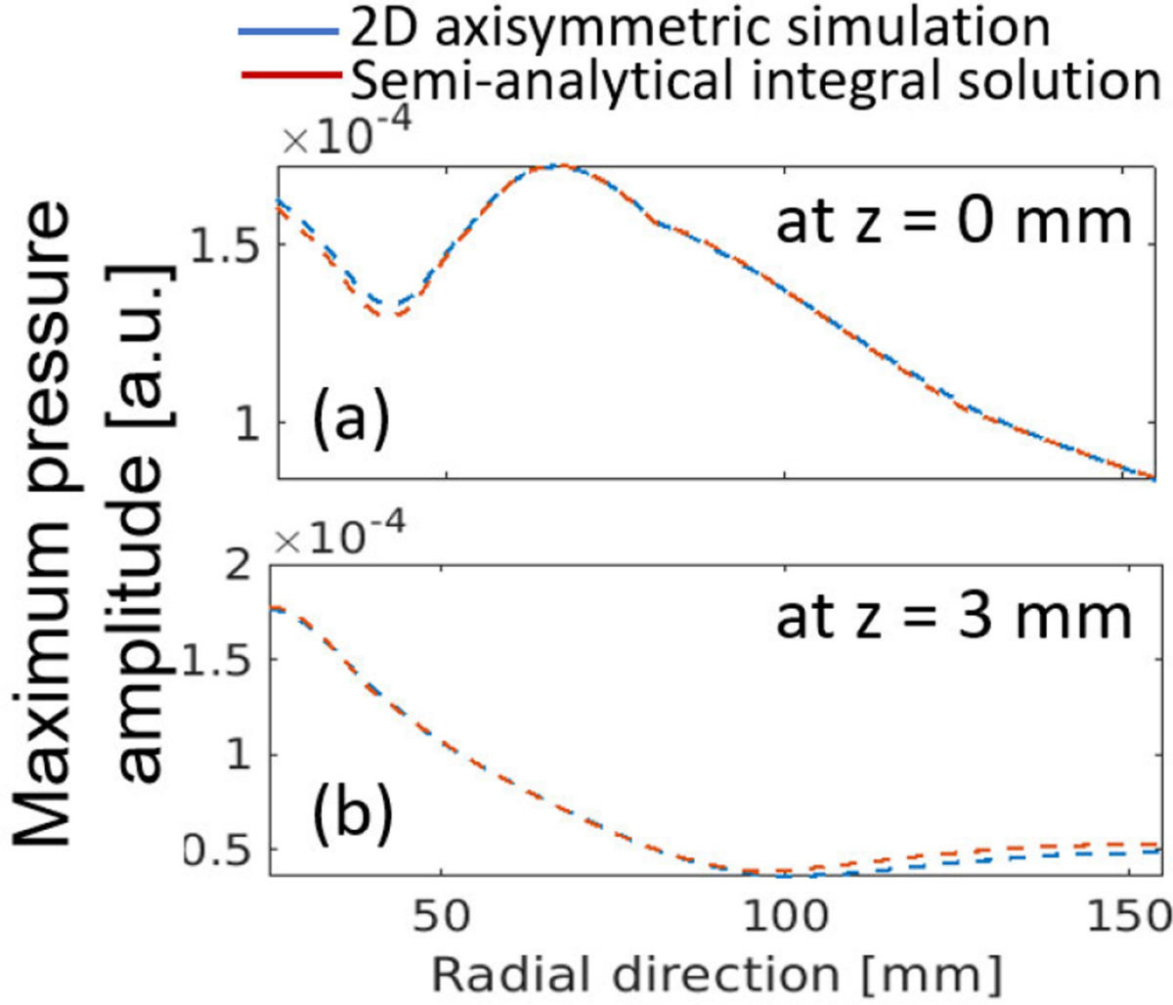
Maximum pressure amplitude profiles for 2-D axisymmetric simulation and semianalytical integral solution with apodization weight function. Lines along the radial direction were plotted (a) at z=0 mm and (b) at z=3 mm. The excitation pulse used in the computation is shown in Fig. 2(a). Pressure amplitude profiles are reported in the same unit as the those used in Fig. 3(d).

**Fig. 13.**
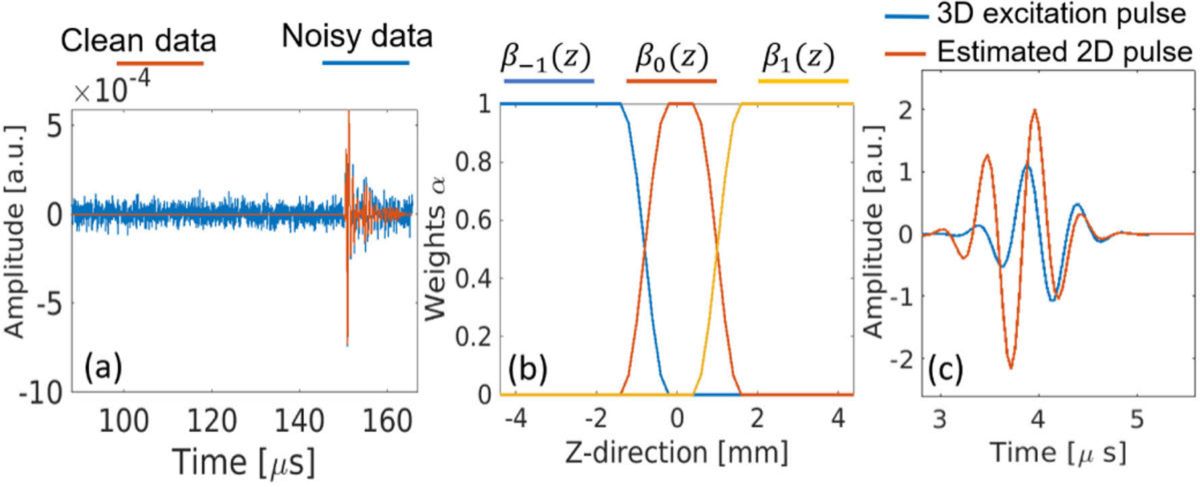
(a) Profiles of the clean and noisy data emitted by the 1th emitter and received by the 512th receiver, for SNR_dB_ = 22 dB. (b) Profile of weights $\beta_{i}(z)$ for combining coarser grid reconstructions from three different ring measurements. (c) Three-dimensional excitation pulse and the estimated effective 2-D excitation pulse.

**TABLE I:** Discretization Parameters of the Virtual Imaging System

Number of emitter/receivers	128/1024
3D Computational grid	[1280, 1280, 216]
Radius of ring-array system	110 mm
Number of ring measurements	3
Scanning interval	1.8 mm
Voxel size	0.2 mm
Time step size	1/25 *μ*s; CFL number=0.3
Simulation time horizon	168 *μ*s
Transducer height	18mm (90 voxels)
Central frequency of source pulse	2 MHz

## Highlights

- A 3-D USCT imaging model that incorporates the transducer’s elevational focusing properties is developed to enable 3-D full waveform inversion (FWI)-based image reconstruction in ring-array USCT.
- The numerical transducer model was quantitatively validated with a semianalytical solution and the acoustic reciprocity principle.
- This work demonstrates the feasibility of incorporating elevational focusing effects on 3-D time-domain FWI for ring-array-based USCT, revealing improved image quality over traditional 2-D methods.

## Footnotes

1 Here, ${\text{ℍ}}^{1}\left({\mathbb{R}}^{3}\times [0,T]\right)$ denotes the space of square integrable functions with square integrable spatiotemporal gradients. Regularity of weak solutions of the wave equation is studied e.g., in [42, Ch 3]. Specifically, a weak solution of the wave equation is such that $p\in L^{2}\left(0,T;{\text{ℍ}}^{1}\left({\mathbb{R}}^{3}\right)\right)$ and $(\partial p/\partial t)\in L^{2}\left(0,T;L^{2}\left({\mathbb{R}}^{3}\right)\right)$, where $L^{2}(0,T;{\text{ℍ}}^{1}\left({\mathbb{R}}^{3}\right)$ and $L^{2}(0,T;L^{2}\left({\mathbb{R}}^{3}\right)$ are the Bochner spaces [43]. It can be shown that if $p\in {\text{ℍ}}^{1}\left({\mathbb{R}}^{3}\times [0,T]\right)$ then p and (∂p/∂t) belongs to the Bochner spaces above.

## Appendix A Publicly Released Datasets

The NBPs and computer-simulated pressure traces used in the case study have been released under Public Domain CC-0 dedication and are available for download from [41].

## Appendix B Modeling of Acoustic Impedance Discontinuity and Attenuation by Apodization Weight Function

In a practical lens-focused transducer, discontinuities in acoustic impedance often occur at the interface between the lens and the propagation medium. Additionally, AA is often present in the lens materials. In this section, the modeling of a lens-focused transducer taking into account the acoustic impedance and AA is presented by introducing an apodization weight function. Additional simulation studies are conducted to demonstrate the use of the apodization weights together with the proposed transducer model in a scenario of practical relevance.

For the transducer in emitter mode, the apodization weight function is applied to the normal velocity, such that (8) is updated as [59] and [60]

(24)
$$v_{⊥}\left(r^{\prime },t\right)=\hat{v}\left(t+\tau \left(r^{\prime }\right)\right)w_{e}\left(r^{\prime }\right)\;\forall r^{\prime }\in \text{Ω}$$

where $w_{e}\left(r^{\prime }\right)$ denotes the apodization weight function at the emitter. It accounts for the attenuation loss due to the AA within the lens and reflection loss due to impedance mismatch as waves travel from the lens to a homogeneous medium. Specifically, $w_{e}\left(r^{\prime }\right)=a\left(r^{\prime }\right)z_{e}\left(r^{\prime }\right)$, where $a\left(r^{\prime }\right)$ is the attenuation factor, which can be modeled as a function of lens thickness $d\left(r^{\prime }\right)$ and the AA value of the lens using a raybased approximation, and $z_{e}\left(r^{\prime }\right)$ is the transmission coefficient accounting for the reflection loss. Assuming the lens is a fluid medium, the transmission coefficient $z_{e}\left(r^{\prime }\right)$ for the transition from the lens to a water medium can be derived based on the laws of reflection and refraction [60], [80]

(25)
$$z_{e}\left(r^{\prime }\right)=\frac{2c_{0}\rho_{0}\cos\theta_{\text{lens}}}{c_{0}\rho_{0}\cos\theta_{\text{lens}}+c_{\text{lens}}\rho_{\text{lens}}\cos\theta_{0}};\;\text{with}\;\;\frac{\sin\theta_{\text{lens}}}{c_{\text{lens}}}=\frac{\sin\theta_{0}}{c_{0}}$$

where $\rho_{\text{lens}}$ and $\rho_{0}$ represent the density values in the lens and homogeneous medium, respectively. The variables $\theta_{\text{lens}}$ and $\theta_{0}$ are functions of $r^{\prime }$, denoting the incident angle and refraction angle, as shown in Fig 10.

Similarly, for the transducer in receiver mode, (10) is updated as

(26)
$$g_{\text{Ω}}(t)=\iint_{\text{Ω}}w_{r}\left(r^{\prime }\right)p(r,t+\tau (r))dr$$

where $w_{r}\left(r^{\prime }\right)$ is the apodization weight function at the receiver. It is modeled as $w_{r}\left(r^{\prime }\right)=a\left(r^{\prime }\right)z_{r}\left(r^{\prime }\right)$, where $z_{r}\left(r^{\prime }\right)=2-z_{e}\left(r^{\prime }\right)$ accounts for the loss when the wave travels from the propagation medium to the lens.

To validate the proposed apodization weight function, and in particular, the acoustic impedance correction $z_{e}\left(r^{\prime }\right)$, an additional simulation study was conducted. A nonattenuating lens was assumed in this study. A high-resolution 2-D axisymmetric simulation [81] is utilized as a reference. The simulation medium is shown in Fig. 11, where the lens is explicitly constructed within the propagation medium by assigning different SOS values to the lens region. In this case, an acoustic impedance discontinuity is present between the lens and propagation medium, where $c_{\text{lens}}\;\rho_{\text{lens}}=3\;c_{0}\rho_{0}$. A nondelayed excitation source pulse is assigned to each pixel on the backplane. The phase changes are achieved by the higher SOS in the lens region. The generated wavefield data are then compared with the semianalytical integral solution incorporating the apodization weight function (with transmission coefficient only), based on (7), (9), (24), and (25).

The proposed 3-D transducer model exhibits cylindrical symmetry and monopolar characteristics (i.e., no directivity) in the imaging plane. As a result, simulation of such transducer model can be efficiently represented by a 2-D axisymmetric simulation, which can largely reduce computational costs and allows for simulations on a finer grid. The computational simulation domain was a high-resolution 2-D Cartesian grid with 500 × 5600 pixels. Each pixel had a size of 0.04 mm. Accordingly, the transducer was divided into a 1-D array of 450 pixels. To ensure numerical stability, the CFL number was set to 0.2, resulting in a time step size of 2 ns. An apodization layer (see Appendix C-A) was introduced between the lens and the perfectly matched layer (PML) [82] to mitigate strong reflections at the back, top, and bottom edges of the lens due to acoustic impedance mismatches.

Fig. 12 shows the comparison of the generated pressure data by two methods. A reasonably close agreement between the two solutions was observed, demonstrating the effectiveness of the apodization weight function in mitigating the impedance mismatch. However, it is important to clarify that we did not anticipate a perfect match, as the 2-D simulation is a discretized model with small stair-casing errors when representing the continuous curvature of the lens on a discrete grid. In addition, due to the complicated design of transducers, accurate modeling of lens-focused transducers in real applications remains a challenging task. The proposed lens model is still a simplified computational model and does not account for the presence of one or more matching layers typically used in piezo-electric transducers to reduce losses induced by acoustic impedance mismatch. To reduce the modeling errors, the apodization weight function can be treated as an unknown surrogate variable and calibrated using experimental data [30], [79].

## Appendix C Implementation Details of the Case Study

### A. Generation of the Measurement Data

The forward simulation of the USCT measurement data was conducted using k-Wave [65], an open-source toolbox for time-domain ultrasound simulations based on the k-space pseudospectral method. To avoid strong wave reflections and scattering that are due to the use of a truncated thin slab phantom and that are not present in a clinical setting, the computational domain is embedded in an extended domain that includes an apodization layer and PML. In the apodization layer, heterogeneous SOS and density maps of the to-be-imaged object are smoothly apodized to match those of the homogeneous coupling medium (water). This apodization layer is introduced because PML can effectively suppress wave reflection at the boundaries of the computational domain [65], [82] only when acoustic heterogeneities are bounded away from the boundary of the domain. In the case study, the computational domain has a height of 27.2 mm and it is embedded in an extended domain with height 43.2 mm by the addition of the apodization layers (4 mm each) and PMLs (4 mm each). The discretization parameters of the forward wave simulation are reported in Table I. For all reconstruction studies, measurement data were corrupted with additive Gaussian noise ϵ with components that are assumed to be i.i.d. with a zero mean and a standard deviation of 3.25e-4. This results in a SNR of 22 dB, which is the logarithmic ratio between the power of the noiseless signal and the power of the noise. An example of time trace of noisy pressure data is shown in Fig. 13(a).

### B. Multigrid Approach for FWI Reconstruction in 3-D

Details regarding the implementation of the coarse-to-fine grid FWI image reconstruction approach are described below. This multigrid approach employs a lower-resolution, lower-frequency FWI reconstruction to provide an initial guess for the higher-resolution, higher-frequency reconstruction. Doing so, not only circumvent the cycle-skipping phenomenon but also leads to significant computational savings since most FWI iterations are performed on the coarse grid. In fact, when a coarse grid with voxel size three times larger than the fine grid is used, each coarse grid FWI iteration is approximately 81 times less computationally expensive than a fine grid iteration. Notably, this multigrid approach also allows for the use of a larger field of view for the coarse grid reconstruction. As described in (21), the computation of the gradient requires access to the forward and adjoint pressure variables at every voxel in the field of view and at every time step. Therefore, computing the gradient for the entire breast region is unfeasible on the fine grid. As a result, while it is feasible to store all pressure variables within the computational domain for the coarse grid reconstruction, in the fine grid reconstruction, only pressure variables within a thin field of view (less than 1 cm of vertical thickness) close to the imaging plane can be stored for gradient computation.

#### 1) Coarse Grid FWI Reconstruction:

In the first step of the reconstruction, the computational grid used for wave simulations is [480, 480, and 108], with a voxel size of 0.6 mm. The transducer is modeled as 30 voxels in height, and the temporal step size is 0.12 *μ*s. A low-pass Hann window filter with a cutoff frequency of 0.45 MHz was applied to the excitation pulse and measured pressure traces to prevent cycle-skipping. The height of the field of view included the entire computational domain and apodization layers (35.4 mm).

#### 2) Construction of the Initial Guess for Fine Grid Reconstruction:

Three image reconstructions using measurements acquired from three distinct vertical positions of the ring array were performed on the coarse grid to define the initial guess for the fine grid reconstruction. Specifically, for i∈{−1,0,1}, the vertical position of the ring array is denoted by $z_{i}=i\text{Δ}z$, where Δz=1.8mm denotes the distance between consecutive vertical positions of the ring array. The coarse grid SOS estimate using data from the ith ring location is denoted by $c_{i}\left(x,\;y,z\right)$. The initial guess for the fine grid reconstruction was then defined as a weighted sum $c(x,y,z)=\sum_{i\in \{-1,0,+1\}}c_{i}(x,y,z)\beta_{i}(z)$ of the coarse grid SOS estimates $c_{i}\left(x,\;y,z\right)$. The weight functions $\beta_{i}\left(z\right)$, those profiles are shown in Fig. 13(b), form a partition of unity ($\beta_{i}\left(z\right)\geq 0$ and $\sum_{i\in \{-1,0,1\}}\beta_{i}(z)=1$) and are such that $\beta_{i}\left(z_{j}\right)=\delta_{ij}$, where $z_{j}$ denotes the elevation of the jth ring (j∈{−1,0,1}) and $\delta_{ij}$ is a Kronecker delta function.

#### 3) Fine Grid FWI Reconstruction:

In the second step, the computational grid size is [1280, 1280, and 180]. The voxel size (0.2 mm) and temporal step size (0.04 *μ*s) is the same as the one in the forward simulation. A low-pass filter with a cutoff frequency of 1.2 MHz is applied to the excitation pulse and measurement data. The height of the field of view is 8 mm that corresponds to a thin slab of high sensitivity to acoustic heterogeneity centered at the imaging plane (see Fig. 5).

#### 4) Stopping Criteria:

The stopping criteria for this multigrid strategy are defined as follows: The iteration with minimum root-mean-square errors (RMSEs) with the true object was selected in both the coarse and fine grid reconstructions.

It is important to note that stopping rules based on the minimum RMSE are not suitable for practical applications since the true object is unknown. However, the primary focus of this work is forward (transducer) modeling and not the development of a practical image reconstruction method. This simple stopping criteria was adopted for the purpose of eliminating the choice of more sophisticated stopping rules for the 2-D and 3-D studies as a potential confounding factor. By minimizing the RMSE, the comparison of 2-D and 3-D methods could be performed in a fair way in our computer simulation studies where the true objects were known. The exploration of alternative stopping criteria or various optimization methods that may improve image quality is left for future research.

### C. Details on the Reference 2-D FWI Reconstruction

The reference SOS image was reconstructed by 2-D FWI using measurement data acquired at ring 0. The transducers were modeled as point-like sources/receivers in the imaging plane. To mitigate the discrepancy between wave propagation in 2-D and 3-D, an effective excitation pulse was estimated by minimizing the Euclidean norm of the difference between pressure traces measured by a receiver diametrically opposed to the source when 2-D and 3-D wave propagation through a homogeneous medium (water). The 3-D excitation pulse and the estimated effective 2-D excitation pulse are shown in Fig. 13(c).

The reference SOS image was obtained by adapting the above FWI multigrid approach to the 2-D case. First, an initial guess is estimated by FWI on a 2-D coarser grid with 480 × 480 pixels, 0.6 mm pixel size, 0.12 *μ*s temporal step size. A low-pass Hann window filter with a cutoff frequency of 0.45 MHz was applied to the excitation pulse and measured pressure traces. The final reconstruction was then performed on a finer grid with 1280 × 1280 pixels, 0.2 mm pixel size, and 0.04 *μ*s temporal step size. A low-pass filter with a cutoff frequency of 1.2 MHz is applied to the excitation pulse and measured pressure traces. The same stopping criteria employed in the 3-D method is used in 2-D.

### D. Computational Time

The majority of the reconstruction time is dedicated to the fine-grid reconstruction process. In the case of 3-D reconstruction, it takes approximately 2.3 days using a single NVIDIA A100 (40 GB memory) GPU on the Delta supercomputer at the University of Illinois at Urbana-Champaign, Urbana, IL, USA. Each iteration of the 3-D fine grid FWI requires around 34 min, while each iteration of the 3-D coarse grid FWI takes about 50 s.

For the 2-D reconstruction, the process takes approximately 4.5 h using a single NVIDIA TITAN X Pascal (12 GB memory) GPU. Each iteration of the 2-D fine grid FWI takes 120 s. On the coarser grid, each iteration takes 16 s.

## References

[1] André M, Wiskin J, and Borup D, “Clinical results with ultrasound computed tomography of the breast,” in Quantitative Ultrasound in Soft Tissues. Cham, Switzerland: Springer, 2013, pp. 395–432.

[2] Roy O. , “Breast imaging using ultrasound tomography: From clinical requirements to system design,” in Proc. IEEE Int. Ultrason. Symp. (IUS), Jul. 2013, pp. 1174–1177.

[3] Guasch L, Calderón Agudo O, Tang M-X, Nachev P, and Warner M, “Full-waveform inversion imaging of the human brain,” NPJ Digit. Med, vol. 3, no. 1, pp. 1–12, Mar. 2020. 32195363 10.1038/s41746-020-0240-8 PMC7060331

[4] Wiskin J, Malik B, Borup D, Pirshafiey N, and Klock J, “Full wave 3D inverse scattering transmission ultrasound tomography in the presence of high contrast,” Sci. Rep, vol. 10, no. 1, pp. 1–14, Nov. 2020. 33214569 10.1038/s41598-020-76754-3 PMC7677558

[5] Wiskin JW , “Imaging of prostate cancer with 3D ultrasound tomography,” Proc. SPIE, vol. PC12038, Apr. 2022, Art. no. PC1203809.

[6] Gierach GL , “Rapid reductions in breast density following tamoxifen therapy as evaluated by whole-breast ultrasound tomography,” J. Clin. Med, vol. 11, no. 3, p. 792, Feb. 2022. 35160244 10.3390/jcm11030792 PMC8836554

[7] Kratkiewicz K, Pattyn A, Alijabbari N, and Mehrmohammadi M, “Ultrasound and photoacoustic imaging of breast cancer: Clinical systems, challenges, and future outlook,” J. Clin. Med, vol. 11, no. 5, p. 1165, Feb. 2022. 35268261 10.3390/jcm11051165 PMC8911419

[8] Littrup PJ , “Multicenter study of whole breast stiffness imaging by ultrasound tomography (SoftVue) for characterization of breast tissues and masses,” J. Clin. Med, vol. 10, no. 23, p. 5528, Nov. 2021. 34884229 10.3390/jcm10235528 PMC8658621

[9] Duric N. , “A novel marker, based on ultrasound tomography, for monitoring early response to neoadjuvant chemotherapy,” J. Breast Imag, vol. 2, no. 6, pp. 569–576, Nov. 2020. 10.1093/jbi/wbaa084 PMC7750888 33385161

[10] Wiskin JW, Borup DT, Iuanow E, Klock J, and Lenox MW, “3-D nonlinear acoustic inverse scattering: Algorithm and quantitative results,” IEEE Trans. Ultrason., Ferroelectr., Freq. Control, vol. 64, no. 8, pp. 1161–1174, Aug. 2017. 28541199 10.1109/TUFFC.2017.2706189 PMC6214813

[11] Wiskin J, Malik B, Natesan R, and Lenox M, “Quantitative assessment of breast density using transmission ultrasound tomography,” Med. Phys, vol. 46, no. 6, pp. 2610–2620, Jun. 2019. 30893476 10.1002/mp.13503 PMC6618090

[12] Pratt RG, Huang L, Duric N, and Littrup P, “Sound-speed and attenuation imaging of breast tissue using waveform tomography of transmission ultrasound data,” Proc. SPIE, vol. 6510, Feb. 2007, Art. no. 65104S.

[13] Wang K, Matthews T, Anis F, Li C, Duric N, and Anastasio MA, “Waveform inversion with source encoding for breast sound speed reconstruction in ultrasound computed tomography,” IEEE Trans. Ultrason., Ferroelectr., Freq. Control, vol. 62, no. 3, pp. 475–493, Mar. 2015. 25768816 10.1109/TUFFC.2014.006788 PMC5087608

[14] Matthews TP, Wang K, Li C, Duric N, and Anastasio MA, “Regularized dual averaging image reconstruction for full-wave ultrasound computed tomography,” IEEE Trans. Ultrason., Ferroelectr., Freq. Control, vol. 64, no. 5, pp. 811–825, May 2017. 28320657 10.1109/TUFFC.2017.2682061 PMC5516530

[15] Duric N, Sak M, and Littrup PJ, “The potential role of the fat–glandular interface (FGI) in breast carcinogenesis: Results from an ultrasound tomography (UST) study,” J. Clin. Med, vol. 10, no. 23, p. 5615, Nov. 2021. 34884317 10.3390/jcm10235615 PMC8658427

[16] Duric N. , “Using whole breast ultrasound tomography to improve breast cancer risk assessment: A novel risk factor based on the quantitative tissue property of sound speed,” J. Clin. Med, vol. 9, no. 2, p. 367, Jan. 2020. 32013177 10.3390/jcm9020367 PMC7074100

[17] Bates O. , “A probabilistic approach to tomography and adjoint state methods, with an application to full waveform inversion in medical ultrasound,” Inverse Problems, vol. 38, no. 4, Apr. 2022, Art. no. 045008. 10.1088/1361-6420/ac55ee PMC7616383 39170751

[18] Lucka F, Pérez-Liva M, Treeby BE, and Cox BT, “High resolution 3D ultrasonic breast imaging by time-domain full waveform inversion,” Inverse Problems, vol. 38, no. 2, 2021, Art. no. 025008.

[19] Javaherian A. and Cox B, “Ray-based inversion accounting for scattering for biomedical ultrasound tomography,” Inverse Problems, vol. 37, no. 11, Nov. 2021, Art. no. 115003.

[20] Hormati A, Jovanovic I, Roy O, and Vetterli M, “Robust ultrasound travel-time tomography using the bent ray model,” Proc. SPIE, vol. 7629, Mar. 2010, Art. no. 76290I.

[21] Schreiman JS, Gisvold JJ, Greenleaf JF, and Bahn RC, “Ultrasound transmission computed tomography of the breast,” Radiology, vol. 150, no. 2, pp. 523–530, Feb. 1984. 6691113 10.1148/radiology.150.2.6691113

[22] Carson PL, Meyer CR, Scherzinger AL, and Oughton TV, “Breast imaging in coronal planes with simultaneous pulse echo and transmission ultrasound,” Science, vol. 214, no. 4525, pp. 1141–1143, Dec. 1981. 7302585 10.1126/science.7302585

[23] André MP, Janée HS, Martin PJ, Otto GP, Spivey BA, and Palmer DA, “High-speed data acquisition in a diffraction tomography system employing large-scale toroidal arrays,” Int. J. Imag. Syst. Technol, vol. 8, no. 1, pp. 137–147, Jan. 1997.

[24] Duric N. , “Detection of breast cancer with ultrasound tomography: First results with the computed ultrasound risk evaluation (CURE) prototype,” Med. Phys, vol. 34, no. 2, pp. 773–785, Jan. 2007. 17388195 10.1118/1.2432161

[25] Song J. , “Design and implementation of a modular and scalable research platform for ultrasound computed tomography,” IEEE Trans. Ultrason., Ferroelectr., Freq. Control, vol. 69, no. 1, pp. 62–72, Jan. 2022. 34410922 10.1109/TUFFC.2021.3105691

[26] Stotzka R, Müller TO, Schlote-Holubek K, and Gemmeke H, “Ultrasound computer tomography for breast cancer diagnosis,” Technol. Health Care, vol. 12, no. 2, pp. 179–182, Jul. 2004.

[27] Gemmeke H. , “The new generation of the KIT 3D USCT,” in Proc. Int. Workshop Med. Ultrasound Tomogr, Hopp T, Ed. Speyer, Germany: KIT Scientific Publishing, Nov. 2018, pp. 271–282.

[28] Malik B, Terry R, Wiskin J, and Lenox M, “Quantitative transmission ultrasound tomography: Imaging and performance characteristics,” Med. Phys, vol. 45, no. 7, pp. 3063–3075, Jul. 2018. 29745992 10.1002/mp.12957 PMC6041196

[29] Cudeiro-Blanco J. , “Design and construction of a low-frequency ultrasound acquisition device for 2-D brain imaging using full-waveform inversion,” Ultrasound Med. Biol, vol. 48, no. 10, pp. 1995–2008, Oct. 2022. 35902276 10.1016/j.ultrasmedbio.2022.05.023

[30] Cueto C. , “Spatial response identification enables robust experimental ultrasound computed tomography,” IEEE Trans. Ultrason., Ferroelectr., Freq. Control, vol. 69, no. 1, pp. 27–37, Jan. 2022. 34383648 10.1109/TUFFC.2021.3104342

[31] Duric N. , “Breast imaging with the SoftVue imaging system: First results,” Proc. SPIE, vol. 8675, Mar. 2013, Art. no. 86750K. 10.1117/12.2002513 PMC10079278 37033290

[32] Duric N. , “Clinical breast imaging with ultrasound tomography: A description of the SoftVue system,” J. Acoust. Soc. Amer, vol. 135, no. 4, p. 2155, Apr. 2014.

[33] Song J, Wang S, Zhou L, Peng Y, Ding M, and Yuchi M, “A prototype system for ultrasound computer tomography with ring array,” in Proc. 2nd IET Int. Conf. Biomed. Image Signal Process. (ICBISP), May 2017, pp. 1–4.

[34] Roberts M, Martin E, Brown M, Cox B, and Treeby B, “Transducer module development for an open-source ultrasound tomography system,” in Proc. IEEE Int. Ultrason. Symp. (IUS), Sep. 2021, pp. 1–4.

[35] Mercep E, Herraiz JL, Deán-Ben XL, and Razansky D, “Transmission–reflection optoacoustic ultrasound (TROPUS) computed tomography of small animals,” Light, Sci Appl., vol. 8, no. 1, p. 18, Jan. 2019. 10.1038/s41377-019-0130-5 PMC6351605 30728957

[36] Sandhu GY, Li C, Roy O, Schmidt S, and Duric N, “Frequency domain ultrasound waveform tomography: Breast imaging using a ring transducer,” Phys. Med. Biol, vol. 60, no. 14, pp. 5381–5398, Jul. 2015. 26110909 10.1088/0031-9155/60/14/5381 PMC4902020

[37] Li F, Villa U, Duric N, and Anastasio MA, “Investigation of an elevation-focused transducer model for three-dimensional full-waveform inversion in ultrasound computed tomography,” Proc. SPIE, vol. 12038, Apr. 2022, pp. 206–214.

[38] Poudel J, Forte LA, and Anastasio MA, “Compensation of 3D-2D model mismatch in ultrasound computed tomography with the aid of convolutional neural networks (conference presentation),” Proc. SPIE, vol. 10955, Mar. 2019, Art. no. 1095507.

[39] Martin E, Ling YT, and Treeby BE, “Simulating focused ultrasound transducers using discrete sources on regular Cartesian grids,” IEEE Trans. Ultrason., Ferroelectr., Freq. Control, vol. 63, no. 10, pp. 1535–1542, Oct. 2016. 27541793 10.1109/TUFFC.2016.2600862

[40] Wise ES, Cox BT, Jaros J, and Treeby BE, “Representing arbitrary acoustic source and sensor distributions in Fourier collocation methods,” J. Acoust. Soc. Amer, vol. 146, no. 1, pp. 278–288, Jul. 2019. 31370581 10.1121/1.5116132

[41] Li F, Villa U, and Anastasio M. (2023). 3D Numerical Breast Phantoms and Ring-Array USCT Measurements (3 Rings). [Online]. Available: 10.7910/DVN/8JVLAE

[42] Lions JL and Magenes E, Non-Homogeneous Boundary Value Problems and Applications Volume 1, vol. 181. Berlin, Germany: Springer, 2012.

[43] Evans LC, An Introduction to Stochastic Differential Equations, vol. 82. Providence RI, USA: American Mathematical Society, 2012.

[44] Treeby BE and Cox BT, “Modeling power law absorption and dispersion for acoustic propagation using the fractional Laplacian,” J. Acoust. Soc. Amer, vol. 127, no. 5, pp. 2741–2748, May 2010. 21117722 10.1121/1.3377056

[45] Chen W. and Holm S, “Modified Szabo’s wave equation models for lossy media obeying frequency power law,” J. Acoust. Soc. Amer, vol. 114, no. 5, pp. 2570–2574, Nov. 2003. 14649993 10.1121/1.1621392

[46] Szabo TL, “Causal theories and data for acoustic attenuation obeying a frequency power law,” J. Acoust. Soc. Amer, vol. 97, no. 1, pp. 14–24, Jan. 1995.

[47] Waters KR, Mobley J, and Miller JG, “Causality-imposed (Kramers-Kronig) relationships between attenuation and dispersion,” IEEE Trans. Ultrason., Ferroelectr., Freq. Control, vol. 52, no. 5, pp. 822–823, May 2005. 16048183 10.1109/tuffc.2005.1503968 PMC13557014

[48] Zhang Z, Huang L, and Lin Y, “Efficient implementation of ultrasound waveform tomography using source encoding,” Proc. SPIE, vol. 8320, Feb. 2012, Art. no. 832003.

[49] Moghaddam PP, Keers H, Herrmann FJ, and Mulder WA, “A new optimization approach for source-encoding full-waveform inversion,” Geophysics, vol. 78, no. 3, pp. R125–R132, May 2013.

[50] Krebs JR , “Fast full-wavefield seismic inversion using encoded sources,” Geophysics, vol. 74, no. 6, pp. WCC177–WCC188, Nov. 2009.

[51] Smith SW, von Ramm OT, Kisslo JA, and Thurstone FL, “Real time ultrasound tomography of the adult brain,” Stroke, vol. 9, no. 2, pp. 117–122, Mar. 1978. 644604 10.1161/01.str.9.2.117

[52] Song C, Xi L, and Jiang H, “Liquid acoustic lens for photoacoustic tomography,” Opt. Lett, vol. 38, no. 15, pp. 2930–2933, 2013. 23903182 10.1364/OL.38.002930

[53] O’Neil HT, “Theory of focusing radiators,” J. Acoust. Soc. Amer, vol. 21, no. 5, pp. 516–526, Sep. 1949.

[54] Hansen CH, “Fundamentals of acoustics,” in Proc. Occupational Exposure Noise, Eval., Prevention Control, vol. 1, no. 3. Geneva, Switzerland: World Health Organization, 2001, pp. 23–52.

[55] Guyomar D. and Powers J, “Transient fields radiated by curved surfaces—Application to focusing,” J. Acoust. Soc. Amer, vol. 76, no. 5, pp. 1564–1572, Nov. 1984, doi: 10.1121/1.391467.

[56] Penttinen A. and Luukkala M, “Sound pressure near the focal area of an ultrasonic lens,” J. Phys. D, Appl. Phys, vol. 9, no. 13, pp. 1927–1936, Sep. 1976, doi: 10.1088/0022-3727/9/13/013.

[57] Yan X-H, Zhang Y-P, Liu K-H, and Liu Y, “Numerical calculation of the sound field focused by acoustic lens with an arbitrary axisymmetric sound speed distribution,” IEEE Trans. Ultrason., Ferroelectr., Freq. Control, vol. 54, no. 4, pp. 823–829, Apr. 2007. 17441591 10.1109/tuffc.2007.315

[58] Wu X. and Sherar M, “Theoretical evaluation of moderately focused spherical transducers and multi-focus acoustic lens/transducer systems for ultrasound thermal therapy,” Phys. Med. Biol, vol. 47, no. 9, pp. 1603–1621, May 2002. 12043823 10.1088/0031-9155/47/9/313

[59] Huo Y. and Chen Y, “Simulation of field characteristics of the focused axisymmetrically curved surface transducers,” IEEE Trans. Ultrason., Ferroelectr., Freq. Control, vol. 48, no. 2, pp. 445–451, Mar. 2001. 11370358 10.1109/58.911727

[60] Maréchal P, Levassort F, Tran-Huu-Hue L-P, and Lethiecq M, “Lens-focused transducer modeling using an extended KLM model,” Ultrasonics, vol. 46, no. 2, pp. 155–167, May 2007. 17382986 10.1016/j.ultras.2007.01.006

[61] Köstli KP and Beard PC, “Two-dimensional photoacoustic imaging by use of Fourier-transform image reconstruction and a detector with an anisotropic response,” Appl. Opt, vol. 42, no. 10, pp. 1899–1908, 2003. 12683772 10.1364/ao.42.001899

[62] Ding L, Deán-Ben XL, and Razansky D, “Efficient 3-D model-based reconstruction scheme for arbitrary optoacoustic acquisition geometries,” IEEE Trans. Med. Imag, vol. 36, no. 9, pp. 1858–1867, Sep. 2017. 10.1109/TMI.2017.2704019 28504935

[63] Wang K, Ermilov SA, Su R, Brecht H-P, Oraevsky AA, and Anastasio MA, “An imaging model incorporating ultrasonic transducer properties for three-dimensional optoacoustic tomography,” IEEE Trans. Med. Imag, vol. 30, no. 2, pp. 203–214, Feb. 2011. 10.1109/TMI.2010.2072514 PMC3033994 20813634

[64] Verweij M, Treeby B, Van Dongen K, and Demi L, “Simulation of ultrasound fields,” in Comprehensive Biomedical Physics, Brahme A, Ed. Oxford, U.K.: Elsevier, 2014, pp. 465–500.

[65] Treeby BE and Cox BT, “K-wave: MATLAB toolbox for the simulation and reconstruction of photoacoustic wave fields,” J. Biomed. Opt, vol. 15, no. 2, 2010, Art. no. 021314. 10.1117/1.3360308 20459236

[66] Norton SJ, “Iterative inverse scattering algorithms: Methods of computing Fréchet derivatives,” J. Acoust. Soc. Amer, vol. 106, no. 5, pp. 2653–2660, Nov. 1999.

[67] Plessix R-E, “A review of the adjoint-state method for computing the gradient of a functional with geophysical applications,” Geophys. J. Int, vol. 167, no. 2, pp. 495–503, Nov. 2006.

[68] Pérez-Liva M, Herraiz JL, Udías JM, Miller E, Cox BT, and Treeby BE, “Time domain reconstruction of sound speed and attenuation in ultrasound computed tomography using full wave inversion,” J. Acoust. Soc. Amer, vol. 141, no. 3, pp. 1595–1604, Mar. 2017. 28372078 10.1121/1.4976688

[69] Kundu T, Placko D, Rahani EK, Yanagita T, and Dao CM, “Ultrasonic field modeling: A comparison of analytical, semi-analytical, and numerical techniques,” IEEE Trans. Ultrason., Ferroelectr., Freq. Control, vol. 57, no. 12, pp. 2795–2807, Dec. 2010. 21156375 10.1109/TUFFC.2010.1753

[70] Treeby BE, Jaros J, Rendell AP, and Cox BT, “Modeling nonlinear ultrasound propagation in heterogeneous media with power law absorption using a K-space pseudospectral method,” J. Acoust. Soc. Amer, vol. 131, no. 6, pp. 4324–4336, Jun. 2012. 22712907 10.1121/1.4712021

[71] Samarasinghe P, Abhayapala TD, and Kellermann W, “Acoustic reciprocity: An extension to spherical harmonics domain,” J. Acoust. Soc. Amer, vol. 142, no. 4, pp. EL337–EL343, Oct. 2017. 10.1121/1.5002078 29092587

[72] Li F, Villa U, Park S, and Anastasio MA, “3-D stochastic numerical breast phantoms for enabling virtual imaging trials of ultrasound computed tomography,” IEEE Trans. Ultrason., Ferroelectr., Freq. Control, vol. 69, no. 1, pp. 135–146, Jan. 2022. 34520354 10.1109/TUFFC.2021.3112544 PMC8790767

[73] Taskin U. and van Dongen KWA, “Multi-parameter inversion with the aid of particle velocity field reconstruction,” J. Acoust. Soc. Amer, vol. 147, no. 6, pp. 4032–4040, Jun. 2020, doi: 10.1121/10.0001396. 32611169

[74] Li F, Villa U, Duric N, and Anastasio MA, “3D full-waveform inversion in ultrasound computed tomography employing a ring-array,” Proc. SPIE, vol. 12470, Apr. 2023, Art. no. 124700K. 10.1109/TUFFC.2023.3313549 PMC10775680 37682648

[75] Yuan Y. , “Full-waveform inversion for breast ultrasound tomography using line-shape modeled elements,” Ultrasound Med. Biol, vol. 49, no. 5, pp. 1070–1081, May 2023. 36737306 10.1016/j.ultrasmedbio.2022.12.004

[76] Cottrell JA, Hughes TJ, and Bazilevs Y, Isogeometric Analysis: Toward Integration of CAD and FEA. Hoboken, NJ, USA: Wiley, 2009.

[77] Tromp J, Komatsch D, and Liu Q, “Spectral-element and adjoint methods in seismology,” Commun. Comput. Phys, vol. 3, no. 1, pp. 1–32, 2008.

[78] Schoeder S, Wall WA, and Kronbichler M, “ExWave: A high performance discontinuous Galerkin solver for the acoustic wave equation,” SoftwareX, vol. 9, pp. 49–54, Jan. 2019.

[79] Roy O, Jovanovic I, Duric N, Poulo L, and Vetterli M, “Robust´ array calibration using time delays with application to ultrasound tomography,” Proc. SPIE, vol. 7968, Mar. 2011, Art. no. 796806.

[80] Dukhin AS and Goetz PJ, “Fundamentals of acoustics in homogeneous liquids: Longitudinal rheology,” in Studies in Interface Science, vol. 24. Amsterdam, The Netherlands: Elsevier, 2010, pp. 91–125.

[81] Treeby BE, Wise ES, Kuklis F, Jaros J, and Cox BT, “Nonlinear ultrasound simulation in an axisymmetric coordinate system using a K-space pseudospectral method,” J. Acoust. Soc. Amer, vol. 148, no. 4, pp. 2288–2300, Oct. 2020, doi: 10.1121/10.0002177. 33138501

[82] Berenger J-P, “A perfectly matched layer for the absorption of electromagnetic waves,” J. Comput. Phys, vol. 114, no. 2, pp. 185–200, Oct. 1994.

